# First evidence of convergent lifestyle signal in reptile skull roof microanatomy

**DOI:** 10.1186/s12915-020-00908-y

**Published:** 2020-11-30

**Authors:** Roy Ebel, Johannes Müller, Till Ramm, Christy Hipsley, Eli Amson

**Affiliations:** 1 Museum für Naturkunde, Leibniz Institute for Evolution and Biodiversity Science, Berlin, Germany; 2 Institute for Biology, Faculty of Life Sciences, Humboldt-Universität zu Berlin, Berlin, Germany; 3 School of BioSciences, The University of Melbourne, Parkville, Victoria 3052 Australia; 4 Sciences Department, Museums Victoria, Carlton, Victoria 3053 Australia

**Keywords:** Convergent evolution, Bone, Microanatomy, Skull roof, Fossorial, Lepidosaur

## Abstract

**Background:**

The study of convergently acquired adaptations allows fundamental insight into life’s evolutionary history. Within lepidosaur reptiles—i.e. lizards, tuatara, and snakes—a fully fossorial (‘burrowing’) lifestyle has independently evolved in most major clades. However, despite their consistent use of the skull as a digging tool, cranial modifications common to all these lineages are yet to be found. In particular, bone microanatomy, although highly diagnostic for lifestyle, remains unexplored in the lepidosaur cranium. This constitutes a key gap in our understanding of their complexly interwoven ecology, morphology, and evolution. In order to bridge this gap, we reconstructed the acquisition of a fossorial lifestyle in 2813 lepidosaurs and assessed the skull roof compactness from microCT cross-sections in a representative subset (*n* = 99). We tested this and five macroscopic morphological traits for their convergent evolution.

**Results:**

We found that fossoriality evolved independently in 54 lepidosaur lineages. Furthermore, a highly compact skull roof, small skull diameter, elongate cranium, and low length ratio of frontal and parietal were repeatedly acquired in concert with a fossorial lifestyle.

**Conclusions:**

We report a novel case of convergence that concerns lepidosaur diversity as a whole. Our findings further indicate an early evolution of fossorial modifications in the amphisbaenian ‘worm-lizards’ and support a fossorial origin for snakes. Nonetheless, our results suggest distinct evolutionary pathways between fossorial lizards and snakes through different contingencies. We thus provide novel insights into the evolutionary mechanisms and constraints underlying amniote diversity and a powerful tool for the reconstruction of extinct reptile ecology.

## Background

As a central aspect to the study of life’s evolutionary history, convergent acquisitions provide fundamental insights into the constraints that shape the phenotype [1, 2]. Highly diagnostic examples of such repeated adaptations can be found in tetrapod bone microanatomy. Lifestyles can thus be inferred from the analysis of trabecular networks [3, 4] and cross-sections of long bone diaphyses [5, 6], ribs [7, 8], and vertebrae [9, 10]. The secondary acquisition of an aquatic lifestyle in tetrapods, for instance, correlates with a non-pathological increase in bone compactness (i.e. osteosclerosis) and morphological robustness (i.e. pachyostosis) [11–13]. Similarly, a fossorial (i.e. burrowing) lifestyle is typically reflected in limb bone cortical thickness [14, 15] but has also been attributed to trabecular anisotropy in mammalian limb bone epiphyses [16, 17]. However, fully fossorial reptiles rarely use their limbs for burrowing and tend to evolve elongate, limb-reduced, head-first burrowing ecomorphs [18–21]. Since this renders the applicability of the established methods impossible, a microanatomical correlate for a fossorial lifestyle in lepidosaurs is yet to be found [8, 22].

Lepidosauria—i.e. lizards, tuatara, and snakes—are the taxonomically and ecologically most diverse non-avian reptile clade [23]. Among other lifestyles, their nearly 11,000 extant species [24] have repeatedly evolved varying degrees of fossoriality [25–27]. A semi-fossorial lifestyle can be found in (1) lacertiform (limbed, lizard-like) taxa occasionally burrowing with their limbs for shelter [28], foraging [29], and reproduction [30] and (2) cryptic serpentiform (limb-reduced, snake-like) taxa that move through substrate of moderate to little resistance, such as leaf litter or loose sand [27]. Contrasting this, fully fossorial lepidosaurs, such as amphisbaenians (“worm lizards”) or blind snakes, spend prolonged periods of time underground [31] and are capable of penetrating substrates of higher resistance [32–34]. The evolution of these limbless, fully fossorial ecomorphs in nearly all major lepidosaur clades is a textbook example of convergence [35–37]. It thus appears particularly desirable to identify a microanatomical lifestyle correlate.

We focused our investigation on the lepidosaur cranium, which is exposed to pronounced strain during head-first burrowing [25]. Although it may potentially provide valuable insight into the constraints that repeatedly shaped fossorial ecomorphs, skull roof compactness has never been systematically quantified in lepidosaurs of different ecologies. This poses a key gap in our understanding of their complexly interwoven ecology, morphology, and evolution. In order to bridge this gap, we reconstructed the acquisition of a fully fossorial lifestyle in 2813 lepidosaurs and assessed the skull roof compactness from microCT cross-sections in a representative subset (*n* = 99). Our dataset also comprises the enigmatic stem-amphisbaenian †*Cryptolacerta hassiaca* (Messel, Eocene of Germany) and four exceptionally well-preserved fossil rhineurids as early representatives of crown amphisbaenians. Alongside skull roof compactness, we tested five macroscopic morphological traits for a lifestyle signal and convergence. In accordance with previous, mostly qualitative mention in a limited number of taxa [18, 25, 32, 38–40], we expect that a fully fossorial lifestyle will be associated with the convergent acquisition of a thick and osteosclerotic (i.e. compact) skull roof with strongly overlapping bones, elongated cranial and parietal proportions, and a small skull diameter. Alternatively, the absence of both a lifestyle signal and a convergent evolution of these traits would suggest that fossoriality had no universal effect on the skull roof of lepidosaurs as a whole. With our study, we set out to contribute to the controversial discussion regarding the ecological origin of ancient amniote lineages, in particular Serpentes and Amphisbaenia, and provide a powerful tool for the reconstruction of extinct amniote ecology.

## Results

### Convergence in lifestyle

**Fig. 1:**
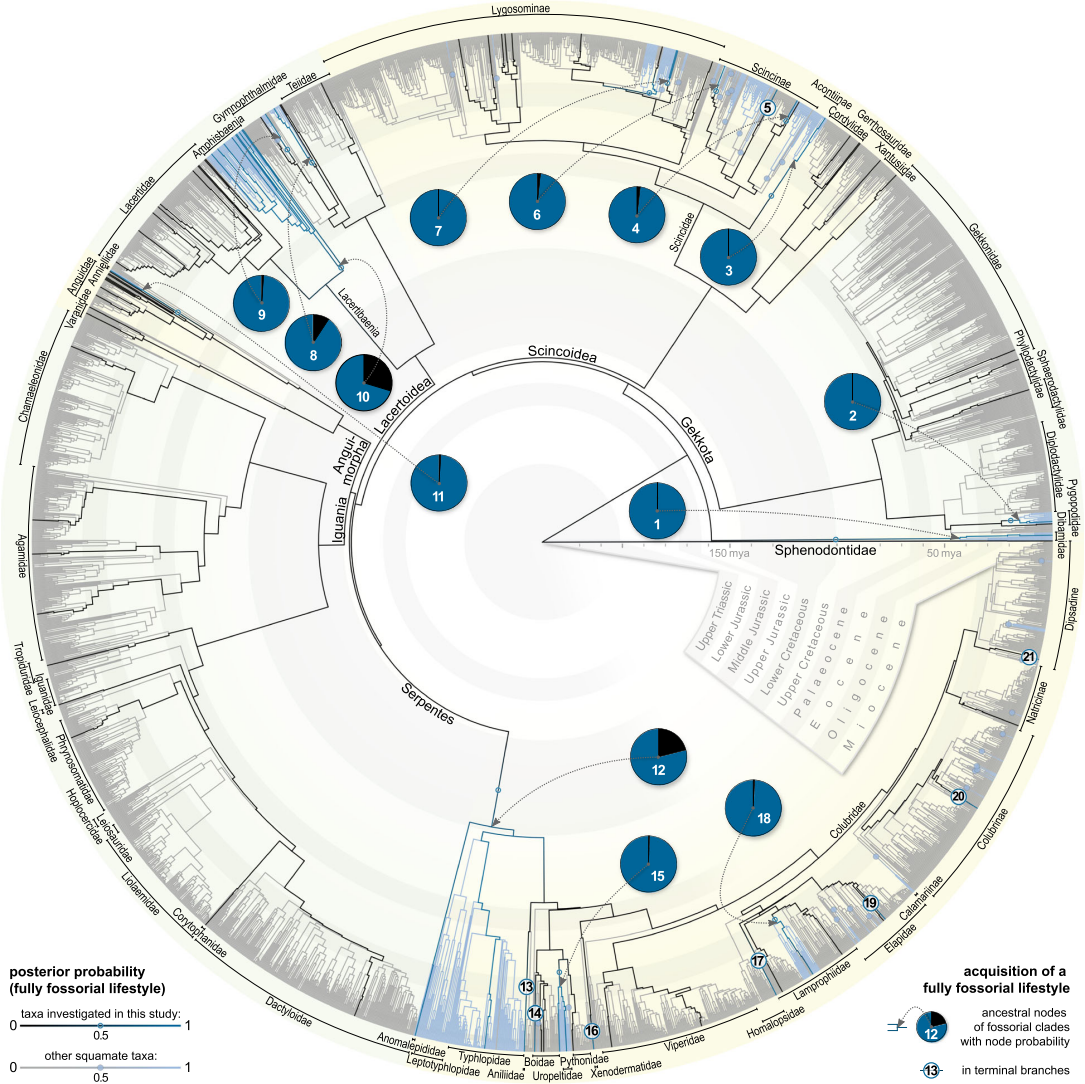
Reconstruction of the acquisition of a fully fossorial lifestyle among lepidosaurs. Non-fossorial lifestyle branches are in grey/black, fossorial ones (as indicated by node posterior probability over 50%) in light blue/dark blue. Phylogeny based on Pyron, Burbrink, and Wiens [41] with a time-calibration in accordance with Ramm et al. [42]. Additional lifestyle data obtained from Bars-Closel et al. [43]. Stochastic character mapping with phytools package [44]. For the taxa further investigated in this study (dark blue/black), the acquisition of a fully fossorial lifestyle is indicated by the numbers 1–21 (see Additional file 1: Table S4 for taxon names). These cover 85% of primary and 35% of secondary acquisitions in the major lepidosaur clades. For a high resolution version of Fig. 1, see Additional file 3

We reconstructed the independent primary acquisition of a fully fossorial lifestyle in 28 lepidosaur lineages. Within the Dibamidae, pygopodid Gekkota, Lacteribaenia, Anguimorpha, and at the base of the Serpentes, we identified one acquisition each. A fully fossorial lifestyle further evolved thrice in the Gymnophthalmidae while the Scincoidea underwent 20 independent acquisitions (Fig. 1; for a high resolution version of Fig. 1, see Additional file 3). Moreover, we reconstructed reversions to a non-fully-fossorial lifestyle as comparatively rare events, only to be observed in the water skink *Eulamprus*, in the long-tailed blind snake *Ramphotyphlops*, and at the base of alethinophidian (i.e. ‘non-blind’) snakes. Within the latter, we found 26 secondary acquisitions of a fully fossorial lifestyle (Fig. 1) and three further reversions.

### Lifestyle signal in skull roof microanatomy and morphology

**Fig. 2:**
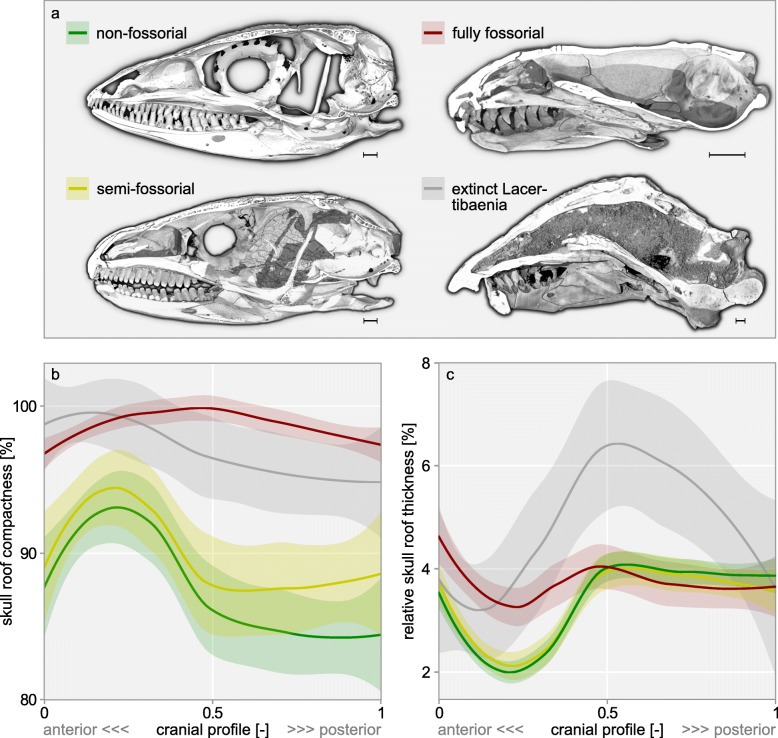
Skull roof structure in 99 lepidosaurs along their cranial profile. Example skulls for lifestyle classes (**a**) as sagittal sections in medial view, scale bar = 1 mm, top left: *Kentropyx altamazonica* ZMB 69836; bottom left: *Egernia kingii* ZMB 21457; top right: *Amphisbaena cubana* ZMB 11034; bottom right: †*Spathorhynchus fossorium* USNM 26317. Loess regressions (lines) with a 95% confidence interval (shaded area) of the compactness (**b**) and relative thickness (**c**) of all sampled specimens are grouped by lifestyle. Fully fossorial taxa consistently exceed non- and semi-fossorial taxa in compactness along the entire cranial profile and in relative thickness in the profile’s anterior half

**Fig. 3:**
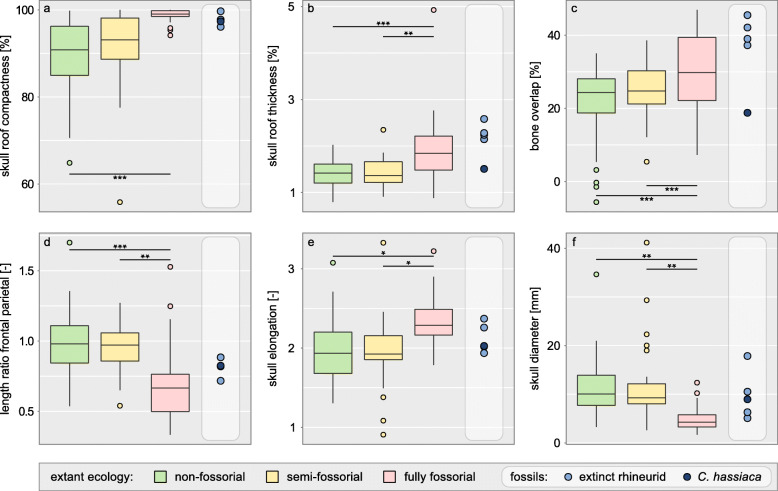
Microanatomical and morphological skull roof traits compared among lifestyle classes. Compactness (**a**), thickness (**b**), overlap (**c**), length ratio of frontal and parietal (**d**), elongation (**e**), and diameter (**f**). Horizontal bars show significant differences as indicated by phylogenetically informed ANOVAs (with adjusted *p* values * ≤ 0.05, ** ≤ 0.01, and *** ≤ 0.001). Comparison with extinct Lacertibaenia in light grey column

Fully fossorial taxa were found to consistently exceed semi- and non-fossorial taxa in skull roof compactness along the whole cranial profile (Fig. 2b). Regarding skull roof thickness, a difference between lifestyles prevails only in the profile’s anterior half (Fig. 2c). Our phylogenetically informed ANOVAs revealed that fully fossorial taxa exceed non-fossorial taxa in skull roof compactness (*p* = 0.0009), thickness (*p* = 0.0003), overlap (*p* = 0.0003), and elongation (*p* = 0.04), while they consistently exhibit a smaller *rfp* (*p* = 0.0006) and skull diameter (*p* = 0.001, Fig. 3a–f). Similarly, fully fossorial taxa were found to exceed semi-fossorial taxa in skull roof thickness (*p* = 0.001), overlap (*p* = 0.001), and elongation (*p* = 0.04) while they exhibit a smaller *rfp* (*p* = 0.001) and diameter (*p* = 0.001, Fig. 3b–f). Contrasting this, skull roof compactness was found not to differ between fully and semi-fossorial taxa (*p* = 0.117, Fig. 3a). We detected no difference regarding any traits between the non- and semi-fossorial classes (all *p* ≥ 0.12). The extinct Lacertibaenia resemble the fully fossorial amphisbaenian crown group regarding their skull roof compactness and, with the exception of †*C. hassiaca*, regarding their skull roof thickness and bone overlap. However, neither skull elongation nor diameter appears to disclose their purported lifestyle (Fig. 3). Although their *rfp* clearly sets the stem-amphisbaenians apart from the Lacertidae, the signal remains ambiguous with regard to the overall dataset.

### Convergence in skull roof microanatomy and morphology

**Fig. 4:**
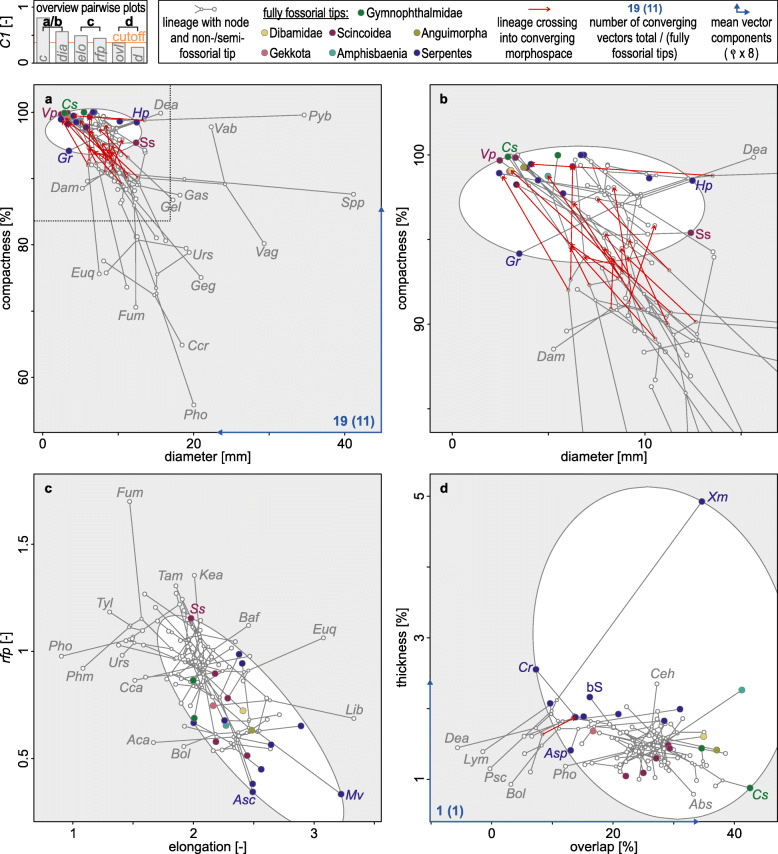
Bivariate morphospaces depicting the convergent evolution of skull roof traits. Converging morphospace ellipsoids are defined by the phenotype of the fully fossorial clades (coloured tips). The convergence index *C5* [45] corresponds to the number of lineages entering this space. As indicated by univariate *C1* (see top left legend box diagram), compactness and diameter (**a**, **b**) are strongly converging—with (**b**) showing an enlarged excerpt of (**a**) in accordance with the dashed line. The ratio of frontal and parietal *rfp* and elongation (**c**) are weakly converging, while overlap and thickness (**d**) are not converging. For key to tip abbreviations, see Additional file 1: Table S6

By means of a univariate implementation of the convergence index *C1*—i.e. the similarity between taxa in comparison with that of their ancestors, proposed by Stayton [45] as an index for the identification and quantification of convergent evolution—we detected a pronounced convergence in skull roof compactness (univariate *C1* = 0.81, *p*_*C1*_ < 0.0001). A moderate to weak convergence was found in skull diameter, elongation, and *rfp* (univariate *C1* ≥ 0.44, *p*_*C1*_ < 0.002). No significant convergence was detected in skull roof thickness and bone overlap (univariate *C1* ≤ 0.36, *p*_*C1*_ > 0.05). The number of convergence events (i.e. univariate *C5* [45];), on the other hand, appears to reflect the evolution of a fully fossorial lifestyle less consistently (Additional file 1: Table S1, Fig. 4) [46, 47], with only moderately informative univariate *C5*-values for elongation and diameter (univariate *C5* ≤ 18, *p*_*C5*_ < 0.0001) and insignificant results for thickness, overlap, and *rfp* (*p*_*C5*_ > 0.15). Only compactness (univariate *C5* = 25, *p*_*C5*_ < 0.0001) defines a morphospace distinguishing all clades with fully fossorial acquisitions (together with a few other clades) from the rest of the sampled lepidosaurs. In concert with the confined morphospace dimensions and the directionality of the mean converging vectors (Fig. 4a), this confirms the exceptional role of skull roof compactness as a convergently evolved fossorial modification.

**Fig. 5:**
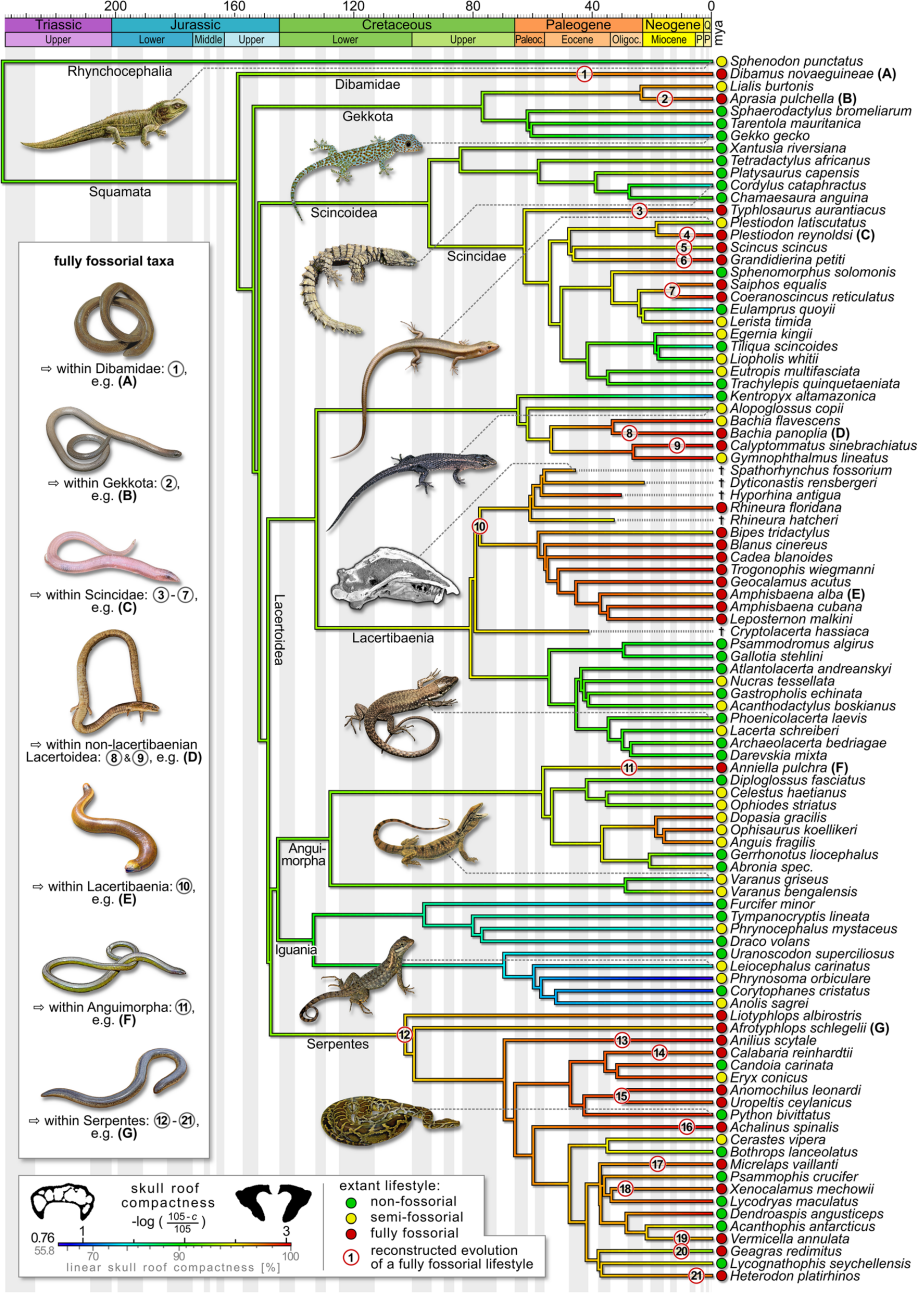
Reconstructed evolution of skull roof compactness in lepidosaurs. Phylogeny based on Pyron, Burbrink, and Wiens [41] with a time-calibration in accordance with Ramm et al. [42] pruned to the sampled taxa. Continuous trait mapping was performed with maximum likelihood (phytools package [44]). Tree was supplemented with stratigraphy (strap package [48]) and visualisation of selected taxa [180–194]. Reconstructed evolution of fossoriality is indicated in accordance with complete phylogeny (Fig. 1)

We consequently reconstructed the evolution of skull roof compactness in order to better understand its interrelation with lifestyle (Fig. 5). The trait increased independently in all lineages that primarily evolved a fully fossorial lifestyle, with the exception of the sandfish *Scincus scincus*. Remarkably, we also observed an increase in skull roof compactness in several semi-fossorial, limb-reduced taxa, such as Burton’s legless lizard *Lialis burtonis*, the dwarf three-toed slider *Lerista timida*, some spectacled lizards of the family Gymnophthalmidae, and glass lizards of the subfamily Anguinae. In a few cases, such as in the Cuban bromeliad geckolet *Sphaerodactylus bromeliarum*, an increase in skull roof compactness was detected despite the absence of a fossorial lifestyle*.* Contrasting this, the majority of non- and semi-fossorial taxa maintained a comparatively low skull roof compactness, with the strictly non-fully-fossorial Iguania exhibiting the lowest skull roof compactness in our dataset. Furthermore, the limbed water skink *Eulamprus quoyii* shows a decrease in skull roof compactness in concert with a reversion to a non-fossorial lifestyle*.* In snakes, lifestyle reversions appear to be less consistently reflected by skull roof microanatomy, with 50% of serpent taxa maintaining a high skull roof compactness after the return to a non- or semi-fossorial lifestyle. On the other hand, in seven out of nine serpent taxa, a secondarily acquired fully fossorial lifestyle is accompanied by a comparatively high skull roof compactness (also mostly exceeding the reconstructed basal serpent condition).

A multivariate assessment revealed that compactness preponderantly explains the observed variation in skull roof structure as the highest loaded variable in *PC1*, which represents 62.1% of the total variance (Additional file 1: Fig. S1, Additional file 1: Table S1). The fully fossorial morphospace is comparatively confined and indicative of a small skull diameter, pronounced elongation, low *rfp*, and high skull roof compactness. Of the 59 extant taxa that fall within its limits, ten are semi-fossorial and 17 are of a non-fossorial lifestyle. It is noteworthy that, of these, 80% and 35% exhibit a serpentiform bauplan, respectively. The extensive morphospaces representing a non- and semi-fossorial lifestyle show a large overlap between one another and with the fully fossorial taxa. With the exception of the comparatively large †*Spathorhynchus fossorium*, all sampled extinct Lacertibaenia were found to fall within the fully fossorial morphospace.

### Size-effect and clade history

In order to investigate the possible effects of constraints beyond fossoriality, we tested skull roof microanatomy and morphology for a size correlation and computed their phylogenetic signal. In the overall dataset, we found that compactness (*p* = 0.002), overlap (*p* = 0.004), *rfp* (*p* = 0.01), and elongation (*p* = 0.002) correlate with size, while only skull roof thickness appears to lack this correlation (*p* = 1.00)_._ However, a within-class analysis revealed no significant differences within the lifestyle subsets (Additional file 1: Table S1), with the exception of compactness and skull elongation in semi-fossorial taxa (0.02 ≤ *p* ≤ 0.04). This suggests that a size effect may not go beyond that of a possible secondary signal in the context of the observed lifestyle-size correlation (Fig. 3f). Comparatively strong phylogenetic signals were detected for skull roof compactness, bone overlap, *rfp*, cranial elongation, and skull diameter (*λ* ≥ 0.66, Additional file 1: Table S1). The phylogenetic signal for skull roof thickness was found to be less pronounced (*λ* = 0.38). In accordance with these observations, we found that lifestyle classes were moderately aggregated within the phylogeny (*RI* = 0.42, *r-PLS* = 0.37). This suggests a possible collateral effect of clade history on cranial microanatomy and morphology despite their clear lifestyle signals.

## Discussion

### Skull microanatomy and morphology as lifestyle correlates

Our findings provide the first comprehensive evidence that fully fossorial lepidosaurs exhibit a thick and highly osteosclerotic (i.e. compact) skull roof, strongly overlapping cranial bones, and a small skull diameter (Fig. 3). Consistent with previous qualitative mention for specific lepidosaur subdivisions [18, 25, 32, 38, 39], the here identified lifestyle signals indicate that these traits may concern the whole diversity of lepidosaurs as fossorial modifications. We also confirm the prevalence of an elongate cranium and low *rfp* (i.e. elongate parietal) in fully fossorial lepidosaurs (Fig. 3) in accordance with a previous large-scale skull morphology study [40]. Of these traits, we identified skull roof compactness, skull diameter, *rfp*, and cranial elongation as convergently evolved in various fully fossorial lepidosaur lineages (Additional file 1: Table S1, Fig. 4). Our findings thus complement the postcranial convergences known in burrowing lepidosaurs [18, 20, 37]. The absence of convergence in skull roof thickness and bone overlap (Fig. 4) suggests that these two traits may reflect lifestyle less consistently.

In accordance with previous descriptions of head-first-burrowing techniques [25, 31, 32], the limitation of a lifestyle signal in skull roof thickness to the anterior half of the cranium (Fig. 2) suggests that burrowing strain is mostly directed at this region. The posterior half of the cranium, on the other hand, exhibits thick layers of epaxial and mandibular muscles in fossorial taxa [39], which may provide additional reinforcement and set limits to bone thickening in favour of a small skull diameter. The latter is constrained through its over-proportionate effect on burrowing work [25]. The epaxial adductor may also explain the parietal elongation, i.e. low *rfp*, in fully fossorial taxa (Fig. 3d), since this muscle (of particular significance to head-first burrowing [25]) attaches along the parietal crest [39]. An elongated parietal further contributes to the cranial elongation typical of fossorial lepidosaurs (Fig. 3e). In analogy to the hydrodynamics of a streamlined body, this modification may reduce drag [64] during a locomotion through a fluid-like, sandy substrate [65]. Elongation may further provide a certain degree of geometry-induced rigidity [66, 67] against axial forces. Additional reinforcement may result from bone overlap (Fig. 3c) since the single overlapping layers contribute to total skull roof thickness and sutures facilitate stress dissipation [68, 69]. A more pronounced overlap may also favour deeper interdigitations and more complex suture lines. Since these features are known in some but not all fossorial lepidosaurs [39, 70], future research should address cranial joint configuration and its biomechanical implications in this context. Cooperative effects on cranial biomechanics could explain why the abovementioned modifications are often observed in concert.

Since the here investigated traits do not discriminate between non- and semi-fossorial taxa (Fig. 3), we conclude that high constraints must act on cranial microanatomy and morphology in association with a fully fossorial lifestyle which appear to differ significantly from those of a semi-fossorial lifestyle. The extensive and strongly overlapping non- and semi-fossorial morphospaces in our PCA confirm that these taxa have a rather generalist skull roof structure (Additional file 1: Fig. S1). This may be explained by the absence of head-first burrowing in the majority of our sampled semi-fossorial taxa, a technique typical of limb-reduced forms [35, 71]. Contrasting this and supporting the high constraints associated with this lifestyle, little disparity was found in the fully fossorial morphospace. However, there is a significant overlap with the non- and semi-fossorial morphospaces. Remarkably, more than half of the non-fully fossorial taxa found in this region are limb-reduced. This suggests that limblessness may co-affect skull roof morphology and microanatomy. While it is tempting to assume that the absence of limbs adds to the constraints acting on the skull by means of its use as a substitute tool, such cranial functions remain under-explored issues [72].

### The exceptional role of skull roof compactness

We demonstrated the exceptional role of skull roof compactness as a lifestyle correlate with its unambiguous lifestyle signal (Fig. 3a), its strong convergence (Fig. 4), and its preponderant contribution to *PC1* (Additional file 1: Table S1). In a reconstruction of trait evolution, we retraced how skull roof compactness independently increased in all lineages that primarily evolved a fully fossorial lifestyle (Fig. 5). As the only exception, *S. scincus* may not be considered head-first-burrowing in sensu stricto since it employs its limbs in a highly specialised swimming motion for the displacement of substrate [65]. A compact skull roof was also found in several semi-fossorial, limb-reduced pygopodid, scincoid, gymnophthalmid, and anguid taxa. Consistent with the insignificant differences between the fully and semi-fossorial classes in our ANOVA (Fig. 3a), this implies that skull roof compactness may be subject to strong constraints associated with even moderate strain exerted on the cranium. The frequent head-first penetration of moderately resistant substrates may provide an explanation. Contrasting this, most non-fossorial and limbed semi-fossorial taxa were found to maintain a low skull roof compactness. Moreover, the case of *E. quoyii* suggests that skull roof microanatomy (in non-serpent lepidosaurs) may return to a less compact state as soon as the constraints of a fossorial lifestyle are abandoned. As a general pattern, these findings demonstrate the interrelation of a primarily evolved fully fossorial lifestyle and a compact skull roof. Despite different underlying biophysics, they parallel the postcranial bone mass increase prevailing in aquatic tetrapods [11, 12]. The knowledge of such microanatomical correlates allows retracing historic lifestyle transitions [7, 13] and may thus contribute to the discussion regarding the ecological origin of various reptilian lineages.

### Amphisbaenian fossorial modifications

We reconstructed the oldest non-serpent acquisition of a fully fossorial lifestyle in the common ancestor of the Amphisbaenia (Fig. 1). A progressively increasing skull roof compactness clearly sets this clade apart from its non- and semi-fossorial sister-taxon, the Lacertidae. Despite varying burrowing techniques [31], we found no differences between keel-, spade-, and round-snouted amphisbaenian skull morphologies. We also found no indication for a repeated evolution of fossorial modifications within the clade [35]. On the contrary, we reconstructed a partial reversion to a less compact skull roof in *Bipes tridactlyus* (Fig. 5). As the only amphisbaenian genus, *Bipes* possesses forelimbs that it uses to excavate a tunnel entry [73], thus possibly reducing cranial strain during this stage of burrowing. Should these aspects be truly interrelated, our findings may support the possibility of a limb re-emergence from a dormant developmental program [74] in this genus as a more parsimonious alternative to a convergent limb reduction in other lineages [35]. On the other hand, the limbed stem-amphisbaenian †*C. hassiaca* demonstrates that fossorial adaptations in the cranium may have evolved early in their clade history and likely predate postcranial modifications, such as limb loss [75]. Although skull roof thickness, elongation, and diameter (Fig. 3) suggest an intermediate lifestyle between lacertid lizards and amphisbaenians, †*C. hassiaca* falls within the fully fossorial morphospace in the PCA (Additional file 1: Fig. S1). In particular, skull roof compactness and *rfp* support its ecological comparability with the amphisbaenian crown group (Fig. 3a, d). Consistent with previous qualitative mention in the literature [70], the fossil rhineurids complement these early fossorial modifications with an increased skull roof thickness and bone overlap (Fig. 3b, c). These findings could suggest that a compactness increase preceded other acquisitions during amphisbaenian evolution. They further demonstrate the applicability of our method on fossil taxa, thus potentially elucidating the evolutionary and ecological history of extinct amniotes.

### Serpent fossorial modifications

The ecological origin of snakes is highly contentious [40, 76, 77]. We reconstructed their transition to a serpentiform bauplan through a fully fossorial lineage in the lower Cretaceous (Fig. 1). Although this contradicts a recent large-scale study [40], a fossorial serpent origin is consistent with previous inferences from cranial and inner ear morphology [77, 78], ecology and habitat use [79], ecomorph evolution [20], Hox gene expression [80], and the fossil record [81, 82]. Fossoriality, however, may be considered an evolutionary dead-end [83], as implied by low diversification rates [43] and rare reversions to other lifestyles in lepidosaurs (Fig. 1). As one explanation, fossorial taxa are constrained to a small skull diameter [20], which limits prey size [84]. The evolution of macrophagy may thus be considered a key innovation that fuelled serpent evolution [85] since the loss of fossoriality in the common ancestor of alethinophidian snakes (Fig. 1). Radiation then produced roughly 3800 extant serpent species [24] of diverse ecologies [43], among them 26 lineages that secondarily acquired a fully fossorial lifestyle—presumably facilitated by ecomorph persistence. However, historical contingency [86] may have created slightly deviating fossorial modifications in these taxa. This is supported by the rather unspecific *C5*-indices (Fig. 4): converging morphospace outer limits are mostly (82%) defined by alethinophidian snakes (and *S. scincus*). When the fully fossorial class is redefined as limbless taxa that primarily evolved a fully fossorial lifestyle (Additional file 1: Fig. S2) [45], the morphospaces become noticeably more confined (by 76.3% ± 12.7%). For the trait pair compactness–diameter (strongly converging according to univariate *C1*), the resulting number of fully fossorial lineages converging (*C5*) in proportion to the total number of fossorial taxa is thus increased from 52% (11 of 21, Fig. 4a, b) to 100% (14 of 14, Additional file 1: Fig. S2). We conclude that there may be deviating constraints that act on the cranial microanatomy and morphology in alethinophidian snakes and *S. scincus*.

Specifically, we observed that 50% of the sampled alethinophidian snakes maintained a highly compact skull roof despite their reversion to a non- or semi-fossorial lifestyle (Fig. 5). These serpents may underlie constraints similar to those discussed for limbless lepidosaurs in general. This finding further complements reports on a uniform bone mass increase indiscriminate of lifestyle in the serpent postcranium [8, 22]. Future research should therefore clarify if systemic bone mass increase [87] may be responsible for this pattern. Secondly, snakes dominate the morphospace indicative of a low skull bone overlap (< 20%) in our convergence analysis, while even distinctly fossorial snakes fall short of the pronounced overlap seen in fossorial non-serpent lepidosaurs (Fig. 4d). A reason may be that alethinophidian snakes employ radically different jaw mechanics in the context of macrophagy, including a prokinetic joint between the nasal, frontal, and prefrontal bones [88]. Although alethinophidian fossorial snakes tend to revert their kinetic skull during the secondary acquisition of a fossorial lifestyle [85], our results demonstrate that this reversion may be incomplete. Thirdly, snakes account for nine of the ten thickest skull roofs (relative to skull size) in the sampled fully fossorial taxa (Fig. 4d), which may be interpreted as an alternative serpent response to a fossorial lifestyle. These altered evolutionary pathways may explain why skull roof thickness and overlap appear to lack a significant convergence in the overall dataset.

### Constraints beyond fossoriality

We considered a possible size effect on skull roof structure but found no correlation between skull diameter and skull microanatomy or morphology within the respective lifestyle classes. As the only exceptions, elongation and compactness were found to be size-correlated in the semi-fossorial class. This, however, should not be overemphasised since this class comprises strongly deviating burrowing techniques, as also indicated by the pronounced microanatomical and morphological disparity found here (e.g. Fig. 3f). Contrasting this, the size-correlations identified in the overall dataset can be explained by the presence of a lifestyle signal in our size proxy (Fig. 3f). The size correlation may thus not exceed the effect of this secondary signal. This, however, does not rule out constraints in miniaturised species, such as in *S. bromeliarum.* As the smallest non-fossorial specimen in our dataset (*SVL* = 24 mm [89], also smaller than 72% of the fossorial taxa), it exhibits the thinnest skull roof in absolute terms (46 μm). This approaches the typical linear osteocyte dimensions in lepidosaurs (5–20 μm [22, 178, 179]). Thinner bone may thus require an acellular organisation, as known from delicate teleost bones [90]. In the skull roof of *S. bromeliarum*, this may pose a lower limit to the inclusion of cavities. Skull roof microanatomy in miniaturised taxa should therefore be further investigated.

Finally, we detected comparatively strong phylogenetic signals for most of the here investigated traits. This may partially result from the detected lifestyle aggregation in the phylogeny (e.g. the absence of fossoriality in the Iguania and Lacertidae but fossorial niche conservatism [91] in the Amphisbaenia). While feeding mechanics appear not to affect the skull morphology in non-serpent fossorial lizards [92], macrophagy and limblessness may constitute serpent-specific constraints (as discussed in the previous subsection). Our findings imply that the lifestyle signal in lepidosaur skull morphology and microanatomy may not be viewed in isolation from constraints associated with clade history and lifestyle aspects beyond fossoriality, such as miniaturisation.

## Conclusion

We identify here a case of convergent evolution that concerns the whole diversity of lepidosaurs: a highly osteosclerotic (i.e. compact) skull roof, small skull diameter, elongate cranium, and low length ratio of frontal and parietal were repeatedly acquired in concert with a fully fossorial lifestyle. Foremost, skull roof compactness plays an exceptional role as a complement to the known microanatomical lifestyle correlates in tetrapods [3–5, 11, 12, 15, 16] that allow a lifestyle reconstruction in extinct taxa. Our results further add to a growing body of evidence for an early evolution of fossorial modifications in the Amphisbaenia and a fossorial origin of snakes. In the latter, we also show how historical contingency associated with limblessness and macrophagy may have altered evolutionary pathways and therefore produced deviating lifestyle responses in cranial microanatomy and morphology. We thus provide insight into the evolutionary mechanisms that repeatedly shaped certain ecomorphs. Beyond the relevance of our findings for the ecological origin of the major lepidosaur lineages, a fossorial lifestyle has likely played a key role in the evolutionary history of various other tetrapod clades [93, 94]. In the context of previously reported cranial modifications [95–97], the skull roof structure in head-first burrowing caecilians and lepospondyls may particularly deserve further investigation. The osteological lifestyle correlate presented here may thus have important implications for reconstructing lifestyle transitions and understanding their impact on the morphology and macroevolution of tetrapods in general.

## Methods

### Specimens and lifestyle

We sampled cranial μCT scans of 99 lepidosaur species from 13 museum collections (Additional file 1: Tables S2 and S3). An assessment of ossification level and suture closure [98] ensured that no juvenile specimens were used in the analysis. Data on taxon lifestyle was obtained from records in the literature (Additional file 1: Table S2) [23, 27, 28, 49–63, 89, 99–155]. In accordance with previous studies [40, 43, 78], we recognised three lifestyle classes defined by the degrees of fossoriality. As fully fossorial, we considered those species for which active head-first burrowing and a preponderant amount of time spent underground were reported. Species capable of excavating a burrow but most often seen above ground were considered as semi-fossorial. This class also includes limb-reduced taxa that may move through a substrate that offers moderate resistance to displacement [27], such as leaf-litter. Third and most common among lepidosaurs [156], a non-fossorial lifestyle was recognised if a burrowing behaviour had not been reported for the species in question. It is noteworthy that this class also comprises limb-reduced taxa, such as grass-swimmers, that may move through microhabitats providing minor resistance to displacement [27]. Our data set thus comprises 38 non-fossorial, 24 semi-fossorial, and 32 fully fossorial species. Furthermore, we sampled exceptionally preserved fossils representing four extinct rhineurids and the stem-amphisbaenian †*C. hassiaca*. A complete list of the sampled specimens, collection numbers, and lifestyle references can be found in Additional file 1: Table S2.

### Cross-section extraction and overall skull measurements

**Fig. 6:**
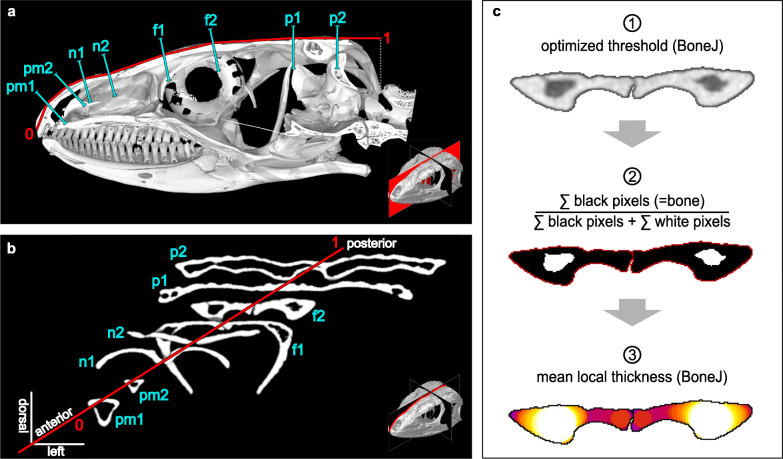
Quantification of microanatomical traits. Cranial profile (red) of a lacertid lizard with skull roof cross sections (turquoise) through premaxilla (pm1/2), nasal (n1/2), frontal (f1/2), and parietal bones (p1/2). Medial view (**a**) of a sagittal section, isolated cross-sections (**b**), and single cross section (f2) as processed with ImageJ and BoneJ (**c**) to measure compactness and thickness. Example skulls: *Gallotia stehlini* ZMB 29084 (**a**), *Darevskia mixta* ZMB 44583 (**b**, **c**)

Cranial μCT scans were mostly acquired at the CT- & visualisation Lab, Museum für Naturkunde, Leibniz Institute for Evolution and Biodiversity Science, Berlin, Germany with GE Phoenix Nanotom S (RRID:SCR_017995) and Yxlon FF35 CT (RRID:SCR_018208). We further included μCT scans acquired at the Helmholtz Centre Berlin for Materials and Energy and the University of Texas High-Resolution X-ray CT Facility (Additional file 1: Table S2). In accordance with specimen size, scan resolution ranged from 3.0 to 61.2 μm (Additional file 1: Table S2). We focused our investigations on the medial skull roof, i.e. the premaxilla, the nasal, the frontal, and the parietal bones. With the 3D-volume processing software VG-Studio Max 3.3 (RRID:SCR_017997), we extracted two transverse cross-sections from each bone, aligned perpendicular to its dorsal surface (Fig. 6a, b). In order to take into account possible anteroposterior variations within the studied bones, we located these sections at one third and two thirds of the anteroposterior bone length. In the case of the fused frontoparietal in *B. tridactylus*, the corresponding positions were inferred from the average proportions in the amphisbaenian clade. As apparent from a comparison between the work of Gans & Montero [39] and Evans [98], bone proportions in the lepidosaur skull roof may deviate considerably in the anteroposterior dimension (Additional file 1: Fig. S3) [39, 98]. In order to take into account these differences, we recorded each cross-section’s position along an anteroposterior cranial profile. We defined these profiles as a succession of the shortest straight lines between the anterior limit of the premaxilla, the intersections between the dorsomedial bone surface and the eight respective cross-sections, and the posterior limit of the parietal (Fig. 6a, b). We further measured the linear skull dimensions, i.e. length (*l*_cran_), width (*w*_cran_), and height (*h*_cran_) of the cranium and articulated mandible, with the distance tool of VG-Studio Max 3.3 (RRID:SCR_017997). As our fossil specimen of †*C. hassiaca* had experienced a dorsoventral taphonomic compression, its original skull proportions were recovered in accordance with the reconstruction of Müller et al. [75]. However, bone overlap data may have become subject to distortion in the context of taphonomic fragmentation in this specimen.

### Microanatomical and morphological parameters

Using the image analysis software ImageJ 1.52i (RRID:SCR_003070), we binarised greyscale tiffs with the optimise threshold function of the plug-in BoneJ 1.4.3 [157] (Fig. 6c). In a few cases, image noise and resolution required manual thresholding and partial manual segmentation of structures in close proximity to one another. In accordance with previous studies [158], bone compactness [dimensionless] was measured as the ratio between the number of pixels defining bone tissue and the total number of pixels inside the outer bone limits (Fig. 6c). We then plotted a LOESS regression [159] of the compactness values against the cross-section positions along the cranial profile (Fig. 2b). Since compactness appeared to differ consistently between the lifestyles along the entire length of the profile, we computed the arithmetic mean of the compactness (*c*) retrieved from the eight cross-sections of a specimen. In order to assess the overall bone thickness, we computed the mean thickness [mm] using the local thickness function provided with BoneJ 1.4.3 [157] after filling all inner cavities (Fig. 6c). Examples of individual specimen thickness and compactness profiles can be found in Additional file 1: Fig. S5 to S7. In order to infer the anteroposterior bone overlap (*ovl*), we employed the following equation:

1 $$ ovl\left[\%\right]=\frac{\frac{L_A+{L}_B}{2}-\left(\varDelta +\frac{L_A}{6}+\frac{L_B}{6}\right)}{\frac{L_A+{L}_B}{2}}=\frac{2}{3}-\frac{2\varDelta }{L_A+{L}_B}, $$

with *Δ* representing the distance between the posterior cross-section of the anterior neighbouring bone *A* and the anterior cross-section of the posterior neighbouring bone *B*—and not the distance between the neighbouring bone limits—hence enabling to compute *ovl* from the recorded cross-section positions along the cranial profile. The overlap thus considered both the length *L* of the neighbouring bones and their position towards one another. An overlap of 100% corresponds to a fully centred alignment of two roofing bones in the anteroposterior dimension while an overlap of 0% was defined as the anterior limit of *B* sharing an identical position with the posterior limit of *A* on the anteroposterior profile. For an interspecific comparison, we calculated the arithmetic mean of the respective three overlapping regions, i.e. premaxilla-nasal, nasal-frontal, and frontal-parietal. Our assessment did not discriminate between dorsoventral, laterolateral, or interdigitated forms of overlap. Since previous studies reported a rapid evolution of frontal and parietal proportions in fossorial lepidosaur taxa [40], we further computed the length ratio (*rfp*) between these two bones:

2 $$ rfp\left[-\right]=\frac{L_{\mathrm{frontal}}\left[\mathrm{mm}\right]}{L_{\mathrm{parietal}}\left[\mathrm{mm}\right]}. $$

### Size correction and thickness integral

A small body size is believed to be a typical concomitant of a fossorial lifestyle [18, 25]. It follows that (1) size needs to be tested for convergent evolution in accordance with lifestyle; (2) if such correlation is present, an exclusion of the size effect through consideration as a covariate in our models could create spurious effects (non-independence of covariate and independent variable); so (3) the exploration of other means for taking size into consideration would appear desirable in order to allow for an interspecific comparison. Given the various degrees and modes of body elongation [160, 161], snout-vent- or body-length may be questionable descriptors for size comparisons between lepidosaurs, while an estimation of body mass (e.g. as proposed by Feldman et al. [162]) would have added another level of potential error to our considerations.

We therefore investigated the suitability of the cranial dimensions as a reference for size correction. To this end, we tested for deviations in skull proportions between the lifestyle classes with a phylogenetically informed ANOVA using the lm.rrpp and pairwise functions of the rrpp package [47] and a within-group correlation structure based on the optimised Pagel’s lambda value [163]. Lambda was recovered using the functions gls of the nlme package [164] and corPagel of the ape package [165] and the time-calibrated phylogeny [41, 42]. Its branch lengths were scaled according to the recovered lambda value with the rescale function of geiger package [166]. This transformed tree was then used for an rrpp regression with 10,000 permutations. If the recovered lambda was negative or greater than 1, its value was forced to be 0 or 1, respectively (see similar protocol in Amson and Kilbourne [167]). All *p* values were adjusted with the Holm method [168] using the p.adjust function of the built-in stats package.

In this exploration of skull proportions, the ratio of skull width and height was found not to differ (*p* = 0.096) in accordance with lifestyle, thus qualifying skull diameter (*dia*):

3 $$ dia\left[\mathrm{mm}\right]=\sqrt{w_{\mathrm{cran}}\left[\mathrm{mm}\right]\cdotp {h}_{\mathrm{cran}}\left[\mathrm{mm}\right]} $$

as a suitable size descriptor and normalisation variable. The absolute skull roof thickness was consequently converted into a relative value (*d*_rel_) as a percentage of the skull diameter. Given that *d*_rel_ was found to consistently reflect lifestyle in some but not the entire profile length (Fig. 2c), a thickness analysis in sub-regions of the skull roof appeared imperative. To this end, we calculated an integral of relative thickness over the cross-section position along the cranial profile. We did this by computing linear regressions between the relative thickness values, i.e. we interpolated the shortest straight lines between our measurements (Additional file 1: Fig. S3). This integral approach takes into account deviations in anteroposterior roofing bone proportions and positions. Skull bones and sutures are known to interact as an integrated compound in the process of stress dissipation [68, 69]. Since a thickness integral provides information on the skull roof as a functional unit, it may thus deliver more relevant values from a biomechanical perspective than the arithmetic mean. In order to assess where deviations in skull roof thickness may preponderantly occur in accordance with the defined lifestyles, we carried out a phylogenetically informed ANOVA (see above) on thickness profile subsections. The lifestyle classes were found not to differ significantly in the posterior half of their thickness profiles (*p* = 0.79). This appears consistent with the notion that the anterior cranium experiences the greatest strain during a head-first burrowing locomotion while the posterior cranium may be sufficiently stabilised by the well-developed epaxial and mandibular adductor muscles [39]. For all subsequent considerations, we thus employed the integral of *d*_rel_ in the limits from 0 to 0.5:

4 $$ d\left[\%\right]=\underset{0.0}{\overset{0.5}{\int }}{d}_{\mathrm{rel}}, $$

hereinafter referred to as skull roof thickness (*d*) for reasons of convenience (Additional file 1: Fig. S3).

Our exploration of skull proportions also revealed that the lifestyle classes differed significantly in skull length-to-width ratio (*p* = 0.003). In accordance with previous reports on skull elongation as a fossorial modification [18, 40]⁠, we therefore considered cranial elongation (*elo*):

5 $$ elo\left[-\right]=\frac{l_{\mathrm{cran}}\left[\mathrm{mm}\right]}{dia\left[\mathrm{mm}\right]}, $$

i.e. the size-normalised skull length, as a separate trait to be tested for convergent evolution in fossorial taxa. We further performed a phylogenetically informed ANOVA (see above) in order to test our investigated dimensionless traits for a size correlation. We found an overall correlation in the full dataset for *ovl*, *rfp*, *elo*, and *c* (0.002 ≤ *p* ≤ 0.012). However, a within-class analysis revealed no significant differences in the majority of lifestyle subsets (Additional file 1: Table S1). This suggests that the size effect does not go beyond that of a secondary signal in the context of a possible lifestyle-size correlation [18, 25]. We therefore refrained from a size-correction of these traits.

### Phylogeny

We used a large-scale lepidosaur phylogeny based on Pyron, Burbrink, and Wiens [41], which was time-calibrated using a penalised likelihood method in the program treePL [169]. We chose 14 calibration points following Ramm et al. [42]. In addition, we positioned the five extinct Lacertibaenia as proposed by Hipsely and Müller [91] with Mesquite 3.04 (RRID:SCR_017994). Where not otherwise noted, further tree manipulation and analyses were carried out with R 3.6.3 (RRID:SCR_001905). For our phylogenetically informed analyses, we pruned the tree to the sampled taxa with the phytools [44] and ape [165] packages. On the occasion of the absence of a sampled species in the phylogeny, we used a sister-taxon instead.

### Ancestral state and clustering of lifestyle

We reconstructed the ancestral character state for lifestyle using the abovementioned phylogeny and stochastic character mapping based on a Markov chain Monte Carlo algorithm [170] with the make.simmap function of phytools package [44]⁠. To this end, we appended the lifestyle information for 2719 additional taxa from Bars-Closel et al. [43] to our dataset and pruned the tree accordingly. These lifestyles were then binarised to form a fully fossorial class and a class comprising all non-fully-fossorial lifestyles. We subsequently visualised the posterior probability for the acquisition of a fully fossorial lifestyle obtained from 10,000 simulations as a continuous character with the densityMap function of phytools package [44]⁠. A list of the reconstructed acquisitions can be found in Additional file 1: Table S4. In order to quantify homoplasy and clustering of fossoriality on the phylogeny, we computed the Retention Index [46] with the RI function of the phangorn package [171]. Likewise, we carried out a two-block partial least squares analysis with the two.b.pls function of the geomorph package [172]⁠, as proposed by Adams and Collyer [173] for the identification of a correlation between the phylogeny and ecological groups.

### Lifestyle signal and quantification of convergent evolution

In order to detect a potential lifestyle signal in compactness, thickness, overlap, *rfp*, elongation, and diameter, we performed phylogenetically informed ANOVAs (see subsection ‘Size correction and thickness integral’). Class mean values of the quantified microanatomical and morphological traits can be found in Additional file 1: Table S1. For individual specimen measurements, see Additional file 1: Table S5. We then assessed Pagel’s *λ* [174] as a measure of the phylogenetic signal in the investigated traits (phylosig function of phytools package [44]). We further investigated the convergent evolution of skull bone microanatomy and morphology in the context of a fully fossorial lifestyle using the framework of the convevol package [45]. For each trait we calculated the *C1* convergence index and *p* value from 30,000 simulations using a univariate version of the convratsig function. The univariate *C1* quantifies the similarity between taxa in comparison with that of their ancestors [45]. Pronounced similarities between taxa that have derived from very dissimilar lineages thus produce large univariate *C1* values close to a maximum value of 1. Rather than testing for convergence between the individual tips, we clustered the members of each converging fully fossorial clade together based on our reconstruction of ancestral lifestyle (see subsection ‘Phylogeny’). We proceeded accordingly in order to exclude within-clade tests for convergence between ancestrally fossorial taxa and reduce the computational effort. We further computed the number of convergence events (*C5*), defined in this framework as the number of lineages entering a morphospace defined by the phenotype of the fully fossorial clades (Fig. 4), using a univariate version of the convnum function [45]. A bivariate implementation was employed for the visualisation in pairwise plots, which we complemented with the average converging vector components, i.e. the sum of all converging lineages divided by their number. We then compared our findings with a pairwise *C5* visualisation comprising alternative converging morphospaces. To this end, we excluded the alethinophidian snakes, which underwent a secondary acquisition of a fully fossorial lifestyle (Fig. 1), and the limbed *S. scincus* from the list of taxa defining the ellipsoids. Moreover, a multivariate exploration of the data was carried out with a principal component analysis [175] (princomp function of the built-in stats package) of those traits that were found to significantly converge and showed a lifestyle signal (Additional file 1: Fig. S1). Doing this, we refrained from employing a phylogenetically informed approach since the properties of a pPCA are more difficult to interpret than those of an ordinary PCA [176]. We further mapped a maximum likelihood reconstruction of the evolution of compactness with the contMap function of phytools package [44] (Fig. 5). Due to the pronounced aggregation of measurements between 95 and 100%, compactness values were log-transformed in order to make differences within this interval clearer:

6 $$ {c}_{\mathrm{log}}=-\log \left(\frac{105\%-c\left[\%\right]}{105\%}\right). $$

We dilated the conversion function horizontally by 5% (arbitrarily chosen) in order to avoid an asymptote at 100%. A visualisation of the conversion scale and trait distribution is provided in Additional file 1: Fig. S4. Stratigraphy was added to the plot with the geoscalePhylo function of the strap package [48]. Where not otherwise noted, figures were compiled with ggplot2 package [177] and post-processed with InkScape 0.92.4 (RRID:SCR_014479).

## Supplementary Information

**Additional file 1: Fig. S1** to **S7** and **Tables S1** to **S6.**

**Additional file 2.** A spreadsheet with all per slice measurements and cross-section positions.

**Additional file 3.** High resolution version of Fig. 1.

## Data Availability

All μCT scans supporting the conclusions of this article are curated in the public digital collection of the Museum für Naturkunde (MfN), Leibniz Institute for Evolution and Biodiversity Science, Berlin, Germany and accessible at the MfN Data Repository (10.7479/4k4c-yc83). All 2D slices exported from these volumes can be found on Figshare (10.6084/m9.figshare.13084583). Further raw data, such as per slice and per specimen measurements, are provided in Additional files 1 and 2. The univariate implementation of the functions of the R package convevol [45]⁠ is available on GitHub (https://github.com/eliamson/convevol1d).
