# Supplementary material for: First evidence of convergent lifestyle signal in reptile skull roof microanatomy
Source: BMC Biol. 2020 Nov 30;18:185. doi: 10.1186/s12915-020-00908-y (PMC7702674; doi:10.1186/s12915-020-00908-y)
Supplement: Supplementary file 1 — Additional file 1: Fig. S1 to S7 and Tables S1 to S6. [file 12915_2020_908_MOESM1_ESM.pdf]

Supplementary Information for

First Evidence of Convergent Lifestyle Signal in Reptile Skull Roof Microanatomy

by Roy Ebel*, Johannes Müller, Till Ramm, Christy Hipsley, and Eli Amson

*email: royebel@hotmail.de

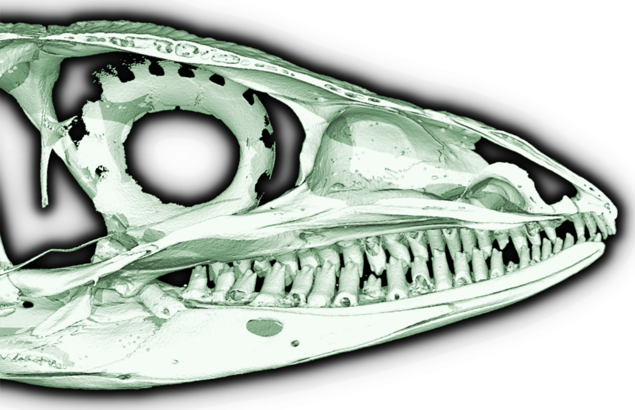

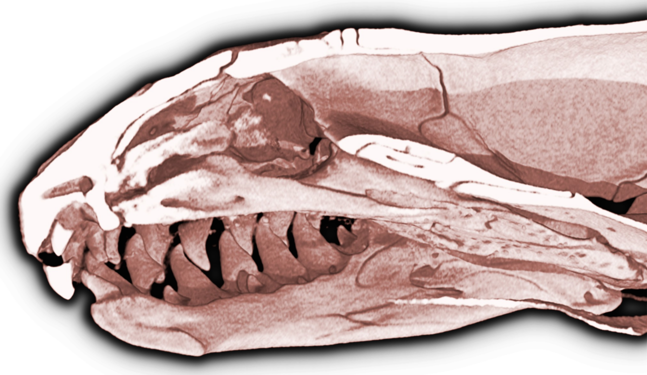

This PDF file includes:

Figure S1. Principal component analysis of microanatomical and morphological traits........................2

Figure S2. Bivariate morphospaces depicting the convergent evolution of skull roof traits...................3

Figure S3. Deviating proportions of frontal and parietal bones..4

Figure S4. Logarithmic compactness conversion with indication of conversion equation.....................4

Figure S5. Individual compactness and thickness profiles of selected Lepidosauria I..........................5

Figure S6. Individual compactness and thickness profiles of selected Lepidosauria II.........................6

Figure S7. Individual compactness and thickness profiles of selected Lepidosauria III........................7

Table S1. Result summary..8

Table S2. Sampled specimens with collection number, scan resolution, and lifestyle...........................9

Table S3. Key to collection numbers from Table S2...12

Table S4. Independent acquisitions of a fully fossorial lifestyle...13

Table S5. Per specimen measurements..14

Table S6. Key to species name abbreviations used in Figure 4 and S2...17

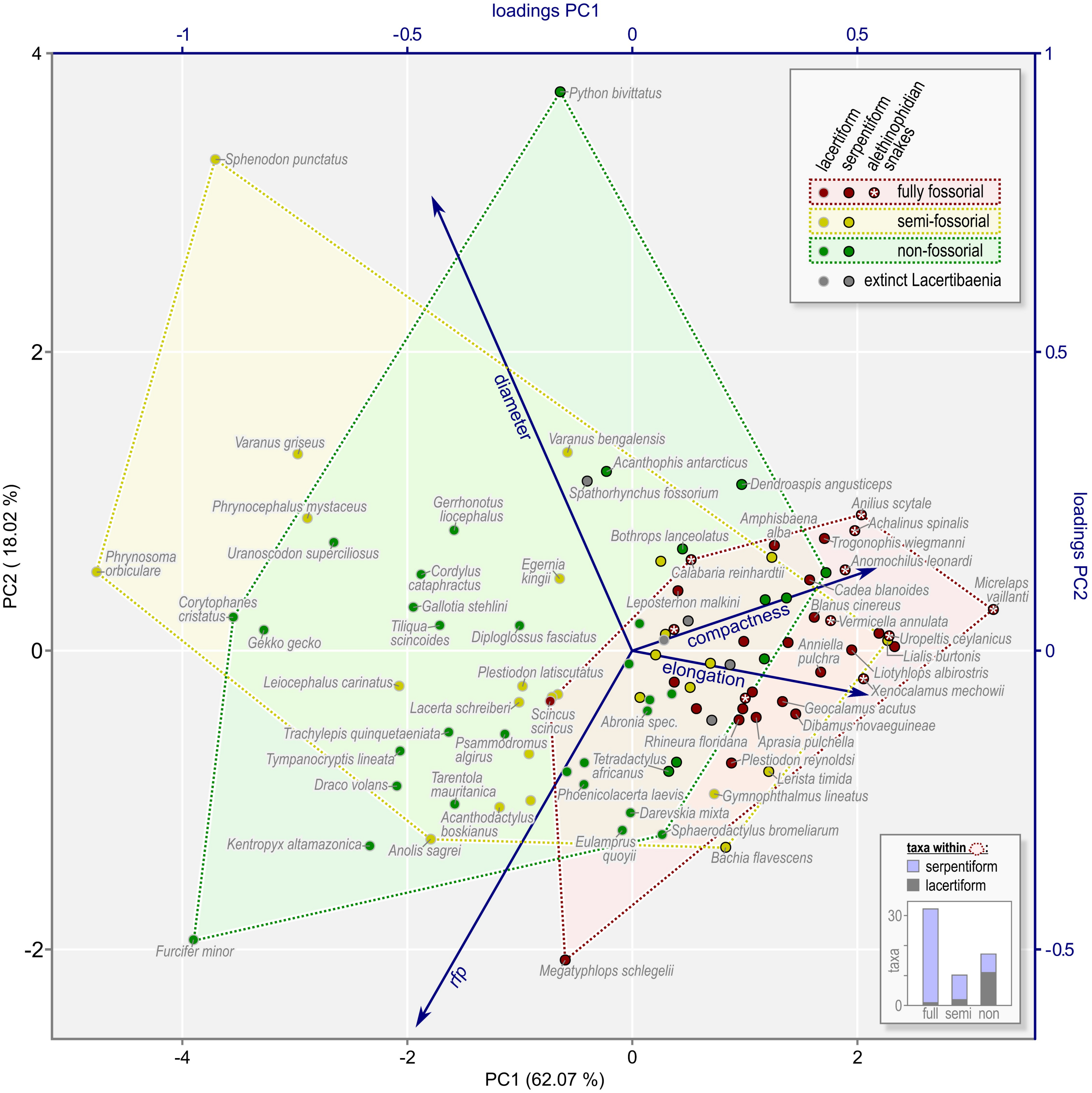

Figure S1. Principal component analysis of microanatomical and morphological traits that were found to significantly converge and showed a significant lifestyle signal: compactness, length ratio of frontal and parietal bones *rfp*, cranial elongation, and skull diameter. Bivariate plot (two first PCs) with convex hulls (dotted lines) indicating the morphospace occupied by each lifestyle. Lacertiform/serpentiform bauplan indicated for all taxa – bottom left inset shows proportions of these bauplans for the 59 extant taxa located inside the region overlapping with the fully fossorial morphospace.

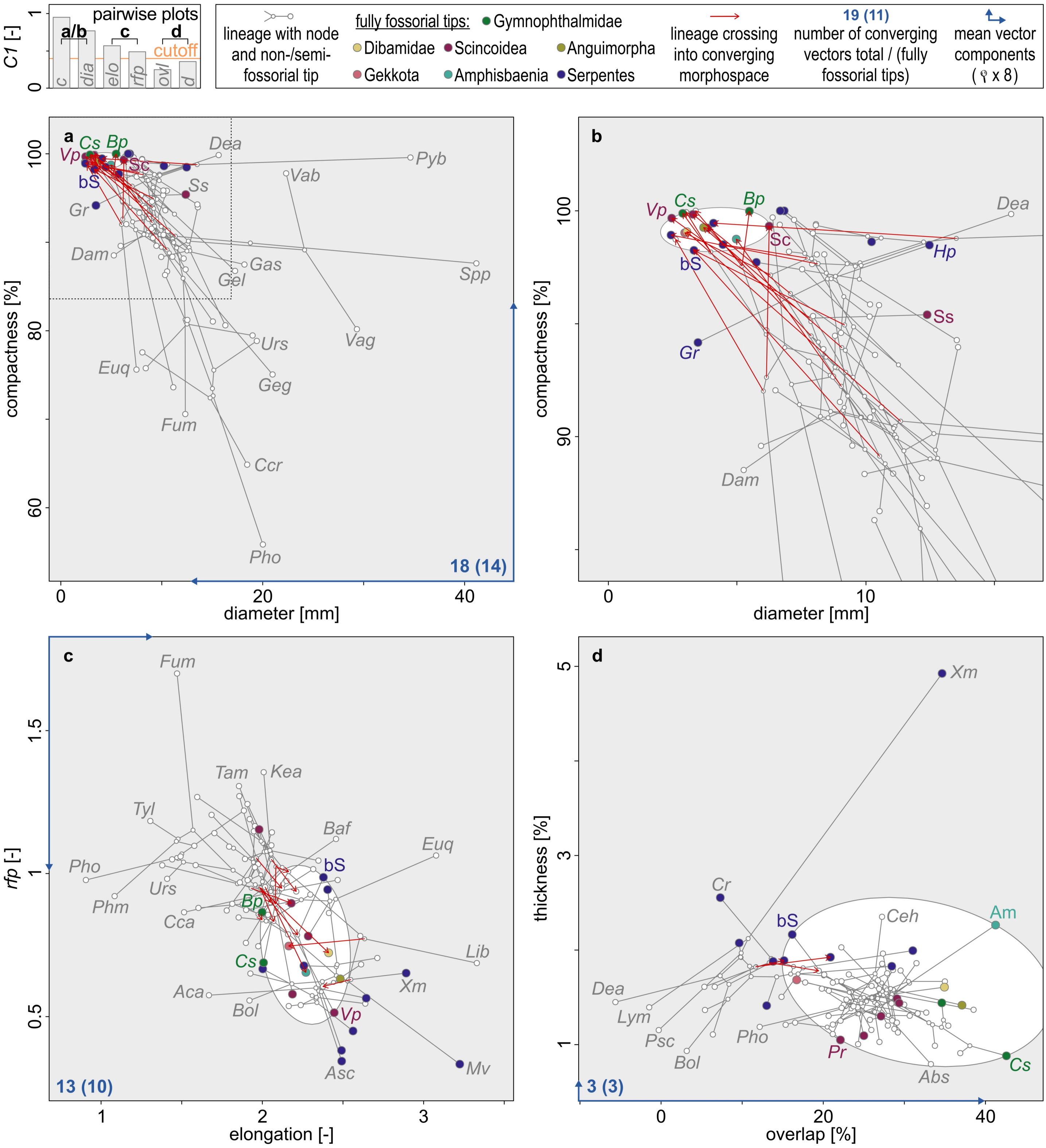

Figure S2. Bivariate morphospaces depicting the convergent evolution of skull roof traits. Different from Figure 4, the converging morphospace ellipsoids are defined by the phenotype of limbless taxa that primarily acquired a fully fossorial lifestyle. The convergence index *C5* [41] corresponds to the number of lineages entering this space. As indicated by univariate *C1* (see top left legend box diagram), compactness and diameter (a, b) are strongly converging – with (b) showing an enlarged excerpt of (a) in accordance with the dashed line. The ratio of frontal and parietal *rfp* and elongation (c) are weakly converging, while overlap and thickness (d) are not converging. Note the consistently greater numbers of morphospace vectors converging to fossorial tips compared to the initial lifestyle coding (Figure 4). For key to tip abbreviations, see Table S6.

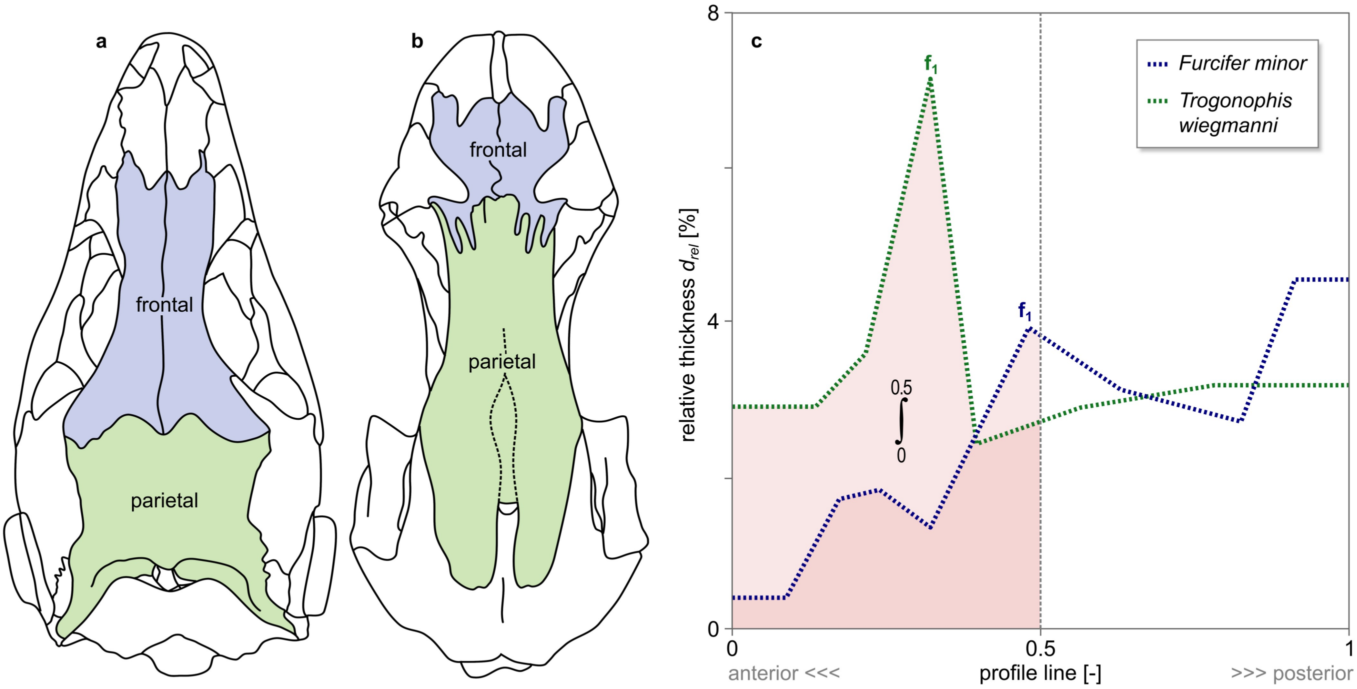

Figure S3. Frontal and parietal bones may have deviating proportions between squamates of different lifestyles. Cranium of a non-fossorial lacertid lizard (a) modified from Evans [81] and a fully fossorial amphisbaenian lizard (b) modified from Gans & Montero [39]. Examples of cranial thickness profiles (c) show that measurements for homologous bones may represent very different subsections of the skull roof as a functional unity. We computed thickness profile integrals in the limits from 0 to 0.5 in order to allow for an unbiased interspecific comparison of the anterior skull roof.

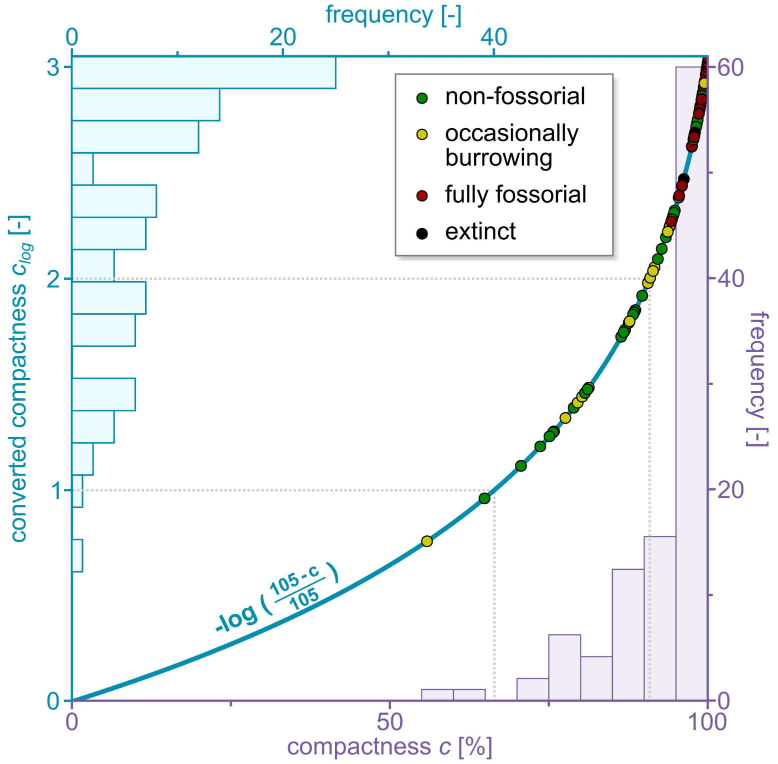

Figure S4. Logarithmic compactness conversion for individual specimens with indication of conversion equation. Compactness distribution appears strongly aggregated between 95 % and 100 % (violet histogram). In this interval, conversion produces a more moderate distribution (light blue histogram) to the benefit of an increased resolution. This allows for a more differentiated visualization of trait evolution when mapped on a phylogeny (Figure 5).

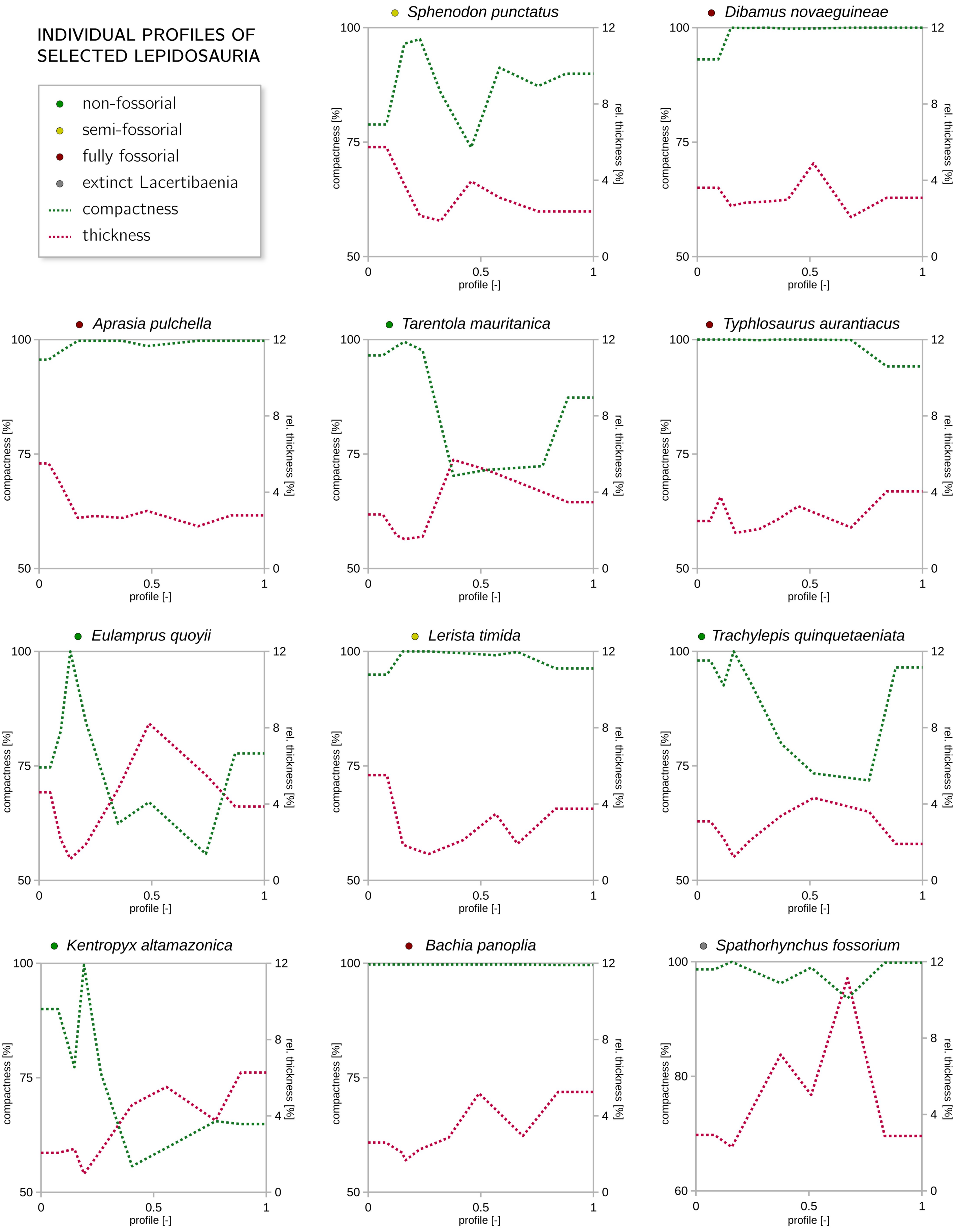

Figure S5. Individual compactness and thickness profiles of selected Lepidosauria I: Sphenodontidae, Dibamidae, Gekkota, Scincoidea, and Lacertoidea 1.

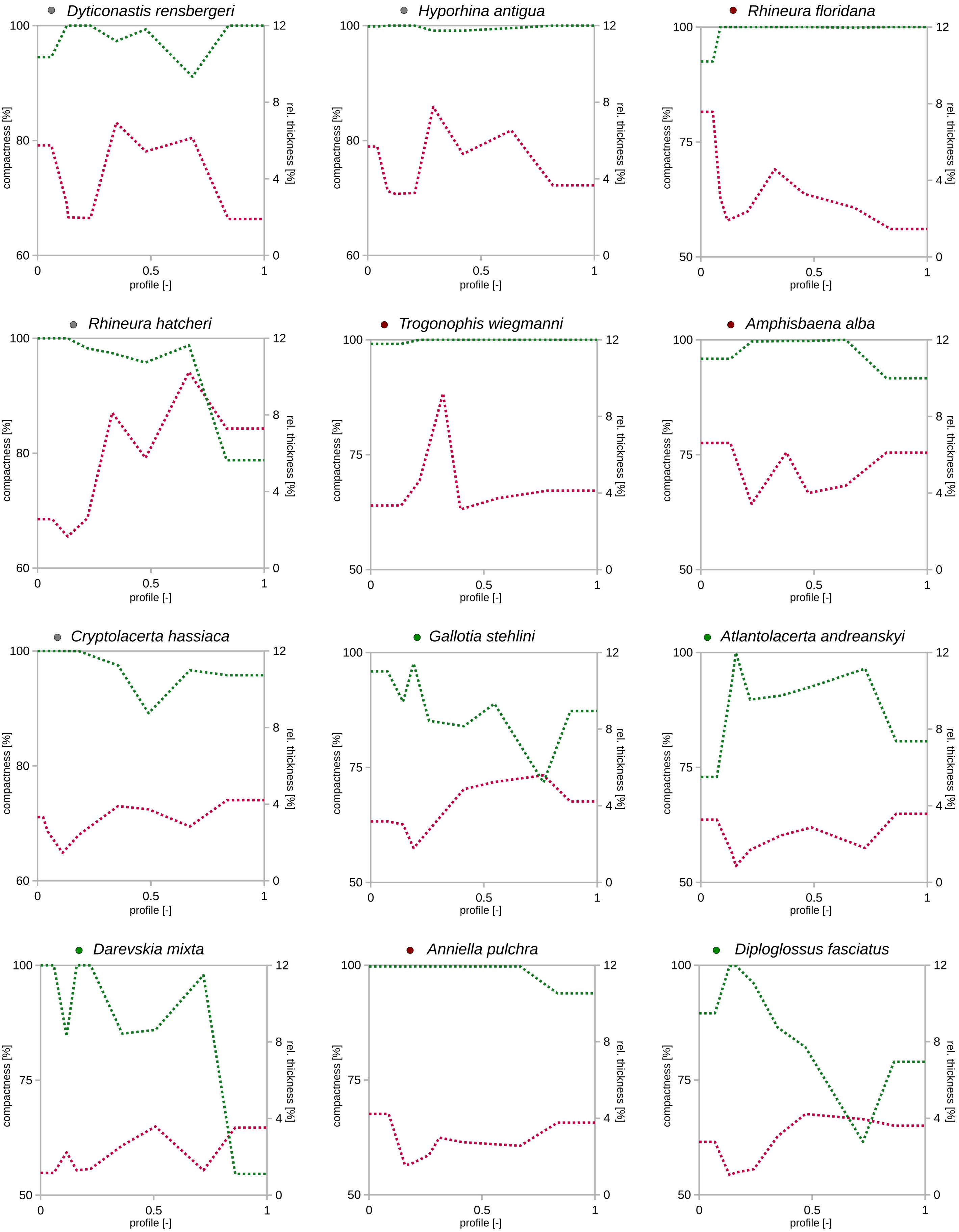

Figure S6. Individual compactness and thickness profiles of selected Lepidosauria II: Lacertoidea 2 and Anguimorpha 1. For legend box, see Figure S5.

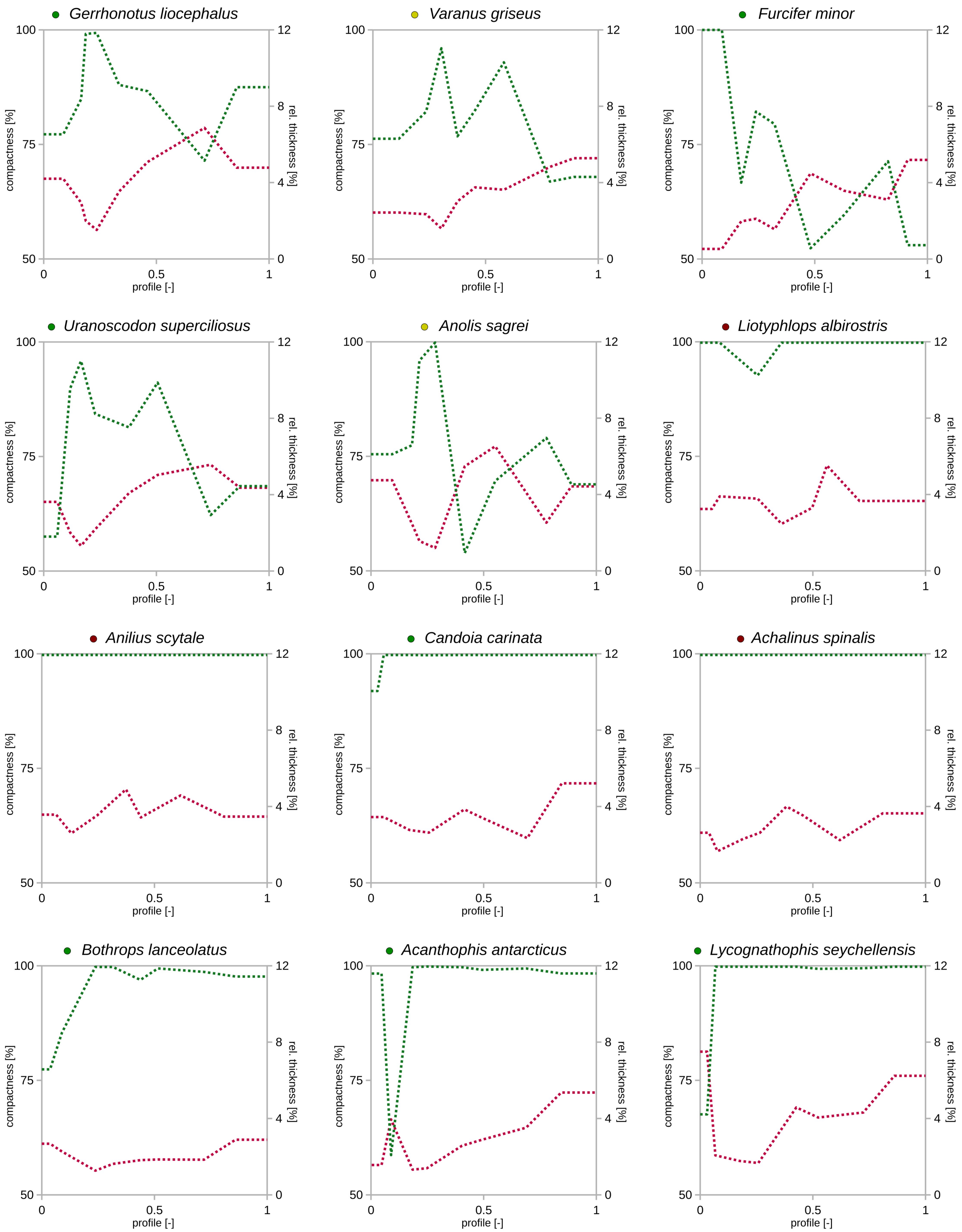

Figure 7. Individual compactness and thickness profiles of selected Lepidosauria III: Anguimorpha 2, Iguania, and Serpentes. For legend box, see Figure S5.

Table S1. Results summary: trait average, phylogenetically informed ANOVAs and PCA, phylogenetic signal and quantification of convergence. Skull roof compactness (*c*), thickness (*d*), bone overlap (*ovl*), ratio of frontal and parietal (*rfp*), cranial elongation (*elo*), and skull diameter (*dia*) with corresponding *p*-values. Skull dimensions length (*l*), height (*h*), and width (*w*) and posterior thickness profile (*d-posterior*). Lifestyles non-fossorial (*non-foss*), semi-fossorial (*occ-burr*), and fully fossorial (*full-foss*) with Retention Index (*RI*, [42]) and partial least squares analysis (*r-PLS*, [43]).

dataset & lifestyle	n_{total}	$n_{non\text{-}foss}$	$n_{occ\text{-}burr}$	$n_{full\text{-}foss}$	$n_{extinct}$	RI_{foss}	$r\text{-}PLS_{foss}$	$p_{r\text{-}PLS}$
	99	36	26	32	5	0.42	0.37	0.005

preliminary phylogenetically informed ANOVAs	$p_{l\sim w}$	$p_{l\sim h}$	$p_{w\sim h}$	p_{l}	$p_{d\text{-}posterior}$
	0.003	0.360	0.055	0.0004	0.79

trait average	class mean & standard deviation	*c* [%]	*d* [%]	*ovl* [%]	*rfp* [-]	*elo* [-]	*dia* [mm]
	non-fossorial	89.0 ± 9.4	1.42 ± 0.29	20.9 ± 10.5	0.97 ± 0.25	1.99 ± 0.42	11.42 ± 5.93
	semi-fossorial	91.0 ± 9.8	1.44 ± 0.33	25.1 ± 7.7	0.96 ± 0.19	1.94 ± 0.46	11.90 ± 8.47
	fully fossorial	98.8 ± 1.4	1.95 ± 0.73	29.6 ± 11.9	0.68 ± 0.27	2.35 ± 0.32	5.13 ± 2.78
	extinct Lacertibaenia	97.8 ± 1.3	2.15 ± 0.39	36.5 ± 10.4	0.81 ± 0.06	2.12 ± 0.18	9.72 ± 5.01

phylogenetically informed ANOVAs		comparison between lifestyle classes	p_c	p_d	p_{ovl}	p_{rfp}	p_{elo}	p_{dia}
		overall comparison	0.0010	0.0004	0.0004	0.0003	0.0207	0.0008
	pairwise	non-fossorial ~ semi-fossorial	0.1175	0.8318	0.2504	0.8529	0.8070	0.4855
	pairwise	non-fossorial ~ fully fossorial	0.0009	0.0003	0.0003	0.0006	0.0384	0.0012
	pairwise	semi-fossorial ~ fully fossorial	0.1168	0.0014	0.0010	0.0014	0.0384	0.0012

phylogenetically informed ANOVAs		log size correlation	p_c	p_d	p_{ovl}	p_{rfp}	p_{elo}
		overall dataset	0.0020	1.0000	0.0036	0.0119	0.0020
	subsets	non-fossorial	0.2328	0.3300	1.0000	1.0000	0.0728
	subsets	semi-fossorial	0.0420	0.3380	0.4014	1.0000	0.0208
	subsets	fully fossorial	1.0000	1.0000	1.0000	0.0897	0.4000

phylo signal	phylogenetic signal in trait evolution	*c*	*d*	*ovl*	*rfp*	*elo*	*dia*
	Pagel's λ	0.88	0.38	0.78	1.00	0.66	0.84
	p_{λ}	$\ll 0.0001$	0.015	$\ll 0.0001$	$\ll 0.0001$	< 0.0001	< 0.0001

convevol package	quantification of convergence	*c*	*d*	*ovl*	*rfp*	*elo*	*dia*
	C1	0.81	0.28	0.36	0.44	0.49	0.56
	cutoff	0.37	0.37	0.37	0.37	0.37	0.37
	p_{C1}	< 0.0001	0.6525	0.0556	0.0018	0.0001	< 0.0001
	C5	25	0	2	0	18	17
	p_{C5}	< 0.0001	1.0000	0.1583	1.0000	< 0.0001	< 0.0001

principal components		principal component analysis	*PC1*	*PC2*	*PC3*	*PC4*	lifestyle signal in *PC1* (phylo. informed ANOVA)	
		variance explained [%]	62.07	18.02	10.82	9.10		
	loadings	*c*	0.544	0.139	0.191	0.805	overall	$p = 0.0001$
	loadings	*rfp*	-0.479	-0.630	-0.335	0.512	non~ semi	$p = 0.6612$
	loadings	*elo*	0.526	-0.074	-0.835	-0.144	non~ full	$p = 0.0003$
	loadings	*dia*	-0.445	0.761	-0.393	-0.262	semi~ full	$p = 0.0006$

Table S2. Sampled specimens with collection number, µCT scan resolution ([µm] = voxel size)**, and lifestyle** (LS, 1 = non-fossorial, 2 = semi-fossorial, 3 = fully fossorial) in accordance with given reference. Where not otherwise noted, µCT scans were carried out at the CT- & Visualization Lab, Museum für Naturkunde, Leibniz Institute for Evolution and Biodiversity Science, Berlin, Germany with GE Phoenix Nanotom S (RRID:SCR_017995) or Yxlon FF35 CT (RRID:SCR_018208).

SPECIES	SPECIMEN	CLADE	HIGHER LEVEL	[µm]	LS	REFERENCE
Sphenodon punctatus	ZMB 13837	Sphenodontidae	Lepidosauria	46.3	2	Dawbin [82]
Dibamus novaeguineae	ZMB 8856	Dibamidae	Squamata	5.8	3	Rieppel [83]
Lialis burtonis	ZMB 10546	Pygopodidae	Gekkota	14.3	2	Shea [84]
Aprasia pulchella	ZMB 25504	Pygopodidae	Gekkota	5.7	3	Shea [84]
Sphaerodactylus bromeliarum	ZMB 42827	Sphaerodactylidae	Gekkota	3.5	1	Peters & Schwartz [70]
Tarentola mauritanica	ZMB 17966	Phyllodactylidae	Gekkota	11.3	1	Johnson, Russell, & Bauer [85]
Gekko gecko	ZMB 48746	Gekkonidae	Gekkota	20.0	1	Subramanean & Vikram Reddy [86]
Xantusia riversiana	ZMB 29899	Xantusiidae	Scincoidea	9.3	1	Fellers & Drost [87]
Tetradactylus africanus	AMNH 57628	Gerrhosauridae	Scincoidea	8.0	1	Mason & Alexander [88]
Platysaurus capensis	ZMB 38563	Cordylidae	Scincoidea	11.4	1	Whiting et al. [89]
Cordylus cataphractus	ZMB 74455	Cordylidae	Scincoidea	14.6	1	Broeckhoven & Mouton [90]
Chamaesaura anguina	ZMB 56421	Cordylidae	Scincoidea	7.5	1	Shine & Wall [91]
Typhlosaurus aurantiacus	ZMB 3936	Scincidae	Scincoidea	4.9	3	Vitt & Caldwell [23]
Plestiodon latiscutatus	ZMB 26508	Scincidae	Scincoidea	10.0	2	Goris [92]
Plestiodon reynoldsi	ZMB 23322	Scincidae	Scincoidea	7.1	3	Pike, Peterman, & Exum [93]
Scincus scincus	ZMB 15318	Scincidae	Scincoidea	13.8	3	Stadler et al. [94]
Grandidierina petiti	ZSM 1620/2010	Scincidae	Scincoidea	8.3	3	Rosa et al. [95]
Sphenomorphus solomonis	ZMB 8803	Scincidae	Scincoidea	9.3	1	Shine & Wall [91]
Saiphos equalis	SAMA R39075	Scincidae	Scincoidea	11.1	3	Shine & Wall [91]
Coeranoscincus reticulatus	SAMA R27452	Scincidae	Scincoidea	20.0	3	Rabosky et al. [96]
Eulamprus quoyii	ZMB 43336	Scincidae	Scincoidea	11.8	1	Law & Bradley [97]
Lerista timida	SAMA R57365	Scincidae	Scincoidea	7.2	2	Robertson & Coventry [98]
Egernia kingii	ZMB 21457	Scincidae	Scincoidea	14.3	2	Chapple [99]
Tiliqua scincoides	ZMB 17061	Scincidae	Scincoidea	16.5	1	Price-Rees, Brown, & Shine [100]
Liopholis whitii	ZMB 29584	Scincidae	Scincoidea	11.3	2	Chapple [99]

Eutropis multifasciata	ZMB 77386	Scincidae	Scincoidea	10.7	2	Catena & Hembree [101]
Trachylepis quinquetaeniata	ZMB 31019	Scincidae	Scincoidea	15.0	1	Spawls et al. [102]
Kentropyx altamazonica	ZMB 69836	Teiidae	Lacertoidea	12.5	1	Vitt et al. [103]
Alopoglossus copii	ZMB 9986	Gymnophthalmidae	Lacertoidea	10.0	2	Hernández-Jaimes, Jerez, & Ramírez-Pinilla [104]
Bachia flavescens	ZMB 8585	Gymnophthalmidae	Lacertoidea	10.0	2	Barros, Herrel, & Kohlsdorf [27]
Bachia panoplia	AMNH R64876	Gymnophthalmidae	Lacertoidea	8.3	3	Barros, Herrel, & Kohlsdorf [27]
Calyptommatus sinebrachiatus	AMNH R138889	Gymnophthalmidae	Lacertoidea	3.3	3	Barros, Herrel, & Kohlsdorf [27]
Gymnophthalmus lineatus	ZMB 28660	Gymnophthalmidae	Lacertoidea	4.9	2	van Buurt [105]
Spathorhynchus fossorium	USNM 26318	Amphisbaenia: Rhineuridae	Lacertoidea	21.5[1]	n/a	[extinct]
Dyticonastis rensbergeri	UCMP 76881[2]	Amphisbaenia: Rhineuridae	Lacertoidea	13.0	n/a	[extinct]
Hyporhina antigua	ZMPU 11390	Amphisbaenia: Rhineuridae	Lacertoidea	5.0	n/a	[extinct]
Rhineura floridana	ZMB 13848	Amphisbaenia: Rhineuridae	Lacertoidea	8.3	3	Vitt & Caldwell [23]
Rhineura hatcheri	CM 15763	Amphisbaenia: Rhineuridae	Lacertoidea	7.5	n/a	[extinct]
Bipes tridactylus	FMNH 265140 CG5528	Amphisbaenia: Bipedidae	Lacertoidea	6.0	3	Vitt & Caldwell [23]
Blanus cinereus	ZMB 1389	Amphisbaenia: Blanidae	Lacertoidea	6.7	3	Vitt & Caldwell [23]
Trogonophis wiegmanni	ZMB 55026	Amphisbaenia: Trogonophidae	Lacertoidea	12.2	3	Vitt & Caldwell [23]
Cadea blanoides	ZMB 4082	Amphisbaenia: Cadeidae	Lacertoidea	11.0	3	Vitt & Caldwell [23]
Geocalamus acutus	ZMB 21941	Amphisbaenia: Amphisbaenidae	Lacertoidea	7.0	3	Vitt & Caldwell [23]
Amphisbaena alba	ZMB 77355	Amphisbaenia: Amphisbaenidae	Lacertoidea	17.7	3	Vitt & Caldwell [23]
Amphisbaena cubana	ZMB 6904	Amphisbaenia: Amphisbaenidae	Lacertoidea	8.3	3	Vitt & Caldwell [23]
Leposternon malkini	MZUSP 13750	Amphisbaenia: Amphisbaenidae	Lacertoidea	12.6	3	Vitt & Caldwell [23]
Cryptolacerta hassiaca	SMF ME 2604[3]	Lacertibaenia	Lacertoidea	12.4	n/a	[extinct]
Psammodromus algirus	ZMB 17931	Lacertidae	Lacertoidea	8.8	1	Martin & López [106]
Gallotia stehlini	ZMB 29084	Lacertidae	Lacertoidea	12.5	1	Carretero et al. [107]
Atlantolacerta andreanskyi	ZFMK 8751	Lacertidae	Lacertoidea	6.1	1	Hoser [108]
Nucras tessellata	ZMB 25635	Lacertidae	Lacertoidea	9.5	2	Cooper & Whiting [109]
Gastropholis echinata	ZMB 11333	Lacertidae	Lacertoidea	16.4	1	Segniagbeto et al. [110]
Acanthodactylus boskianus	ZMB 70859	Lacertidae	Lacertoidea	8.9	2	Zaady & Bouskila [111]
Phoenicolacerta laevis	ZMB 14316	Lacertidae	Lacertoidea	10.4	1	Modrý et al. [112]
Lacerta schreiberi	ZMB 11024	Lacertidae	Lacertoidea	10.7	2	Marco, Díaz-Paniagua, & Hidalgo-Vila [113]

Archaeolacerta bedriagae	ZMB 69157	Lacertidae	Lacertoidea	10.5	1	Bombi et al. [114]
Darevskia mixta	ZMB 44583	Lacertidae	Lacertoidea	7.9	1	Gabelaia, Adriaens, & Tarkhnishvili [115]
Anniella pulchra	ZMB 7843	Anniellidae	Anguimorpha	6.4	3	Vitt & Caldwell [23]
Diploglossus fasciatus	ZMB 1183	Anguidae	Anguimorpha	14.3	1	Ribeiro & Amaral [116]
Ophiodes striatus	ZMB 67020	Anguidae	Anguimorpha	14.5	2	Oliveira [117]
Celestus haetianus	ZMB 52518	Anguidae	Anguimorpha	12.1	2	Thomas [118]
Dopasia gracilis	ZMB 9930	Anguidae	Anguimorpha	12.0	2	Verma [119]
Ophisaurus koellikeri	ZMB 18006	Anguidae	Anguimorpha	10.0	2	de Pous et al. [120]
Anguis fragilis	ZMB 3239	Anguidae	Anguimorpha	9.4	2	Blain, Bailon, & Agustí [121]
Gerrhonotus liocephalus	ZMB 6691	Anguidae	Anguimorpha	15.5	1	Flury [122]
Abronia spec.	ZMB 67130	Anguidae	Anguimorpha	10.4	1	Vitt & Caldwell [23]
Varanus griseus	ZMB 36602	Varanidae	Anguimorpha	61.2	2	Tsellarius & Tsellarius [123]
Varanus bengalensis	ZMB 16299	Varanidae	Anguimorpha	34.8	2	Traeholt [124]
Furcifer minor	ZMB 10411	Chamaeleonidae	Iguania	13.0	1	Randrianantoandro, Andriantsimanarilafy, & Randrianavelona [125]
Tympanocryptis lineata	ZMB 4714	Agamidae	Iguania	10.9	1	Melville & Schulte [28]
Phrynocephalus mystaceus	ZMB 44079	Agamidae	Iguania	17.5	2	Panov, Tsellarius, & Nepomnyashchikh [126]
Draco volans	ZMB 31390A	Agamidae	Iguania	7.8	1	Hairston [127]
Uranoscodon superciliosus	ZMB 30932	Tropiduridae	Iguania	18.5	1	Howland, Vitt, & Lopez [128]
Leiocephalus carinatus	ZMB 78161	Leiocephalidae	Iguania	12.9	2	Kavaliers, Courtenay, & Hirst [129]
Phrynosoma orbiculare	ZMB 37142	Phrynosomatidae	Iguania	14.0	2	Weese [130]
Corytophanes cristatus	ZMB 80461	Corytophanidae	Iguania	17.1	1	Townsend et al. [131]
Anolis sagrei	ZMB 537	Dactyloidae	Iguania	9.1	2	Pacala, Rummel, & Roughgarden [132]
Liotyphlops albirostris	ZMB 9529	Anomalepidae	Serpentes	3.0	3	Rieppel, Kley, & Maisano [133]
Afrotyphlops schlegelii	ZMB 36884	Typhlopidae	Serpentes	9.4	3	Allemand et al. [134]
Anilius scytale	ZMB 1438	Aniliidae	Serpentes	12.5	3	Allemand et al. [134]
Calabaria reinhardtii	ZMB 20308	Boidae	Serpentes	19.4	3	Angelici et al. [135]
Candoia carinata	ZMB 14571	Boidae	Serpentes	11.7	1	Jayne [136]
Eryx conicus	ZMB 1463	Boidae	Serpentes	13.0	2	Pough [137]
Anomochilus leonardi	ZMB 80303	Anomochildiae	Serpentes	6.4	3	Rieppel & Maisano [138]
Uropeltis ceylanicus	ZMB 90312	Uropeltidae	Serpentes	6.7	3	Gower, Captain, & Thakur [139]

Python bivittatus	ZMB 30906	Pythonidae	Serpentes	40.7	1	Sharma & Kandel [140]
Achalinus spinalis	ZMB 27948A	Xenodermatidae	Serpentes	10.7	3	Yamasaki & Mori [141]
Cerastes vipera	ZMB 20862	Viperidae	Serpentes	12.2	2	Ibrahim [142]
Bothrops lanceolatus	ZMB 2946	Viperidae	Serpentes	20.0	1	Gros-Désormeaux et al. [143]
Micrelaps vaillanti	ZMB 22406	Lamprophiidae	Serpentes	5.0	3	Rasmussen [144]
Psammophis crucifer	ZMB 16482	Lamprophiidae	Serpentes	12.5	1	Shine et al. [145]
Xenocalamus mechowii	ZMB 23387	Lamprophiidae	Serpentes	8.7	3	Bates [146]
Lycodryas maculatus	ZMB 19226	Lamprophiidae	Serpentes	14.1	1	Vences et al. [147]
Dendroaspis angusticeps	ZMB 56385	Elapidae	Serpentes	23.2	1	Petras et al. [148]
Acanthophis antarcticus	ZMB 38580	Elapidae	Serpentes	14.0	1	Smith et al. [149]
Vermicella annulata	ZMB 63500	Elapidae	Serpentes	11.0	3	Johnson [150]
Geagras redimitus	MNHN RA 8404	Colubridae	Serpentes	4.3	3	Holm [151]
Lycognathophis seychellensis	ZMB 7617	Colubridae	Serpentes	13.4	1	Bowler [152]
Heterodon platirhinos	ZMB 13871	Colubridae	Serpentes	15.0	3	Edgren [153]

[1] in-plane resolution, voxel size = 21.5 µm x 21.5 µm x 47.2 µm, resulting cross section resolution = 28.7 µm - 33.0 µm
[2] scanned at the University of Texas High-Resolution X-ray CT Facility
[3] scanned at the Helmholtz Centre Berlin for Materials and Energy using a micro-focus X-ray

Table S3. Key to collection numbers from Table S2.

AMNH	American Museum of Natural History, New York, NY, United States of America
CM	Carnegie Museum of Natural History, Pittsburgh, PA, United States of America
FMNH	Field Museum of Natural History, Chicago, IL, United States of America
MNHN	Muséum national d'Histoire naturelle, Paris, France
MZUSP	Museum of Zoology of the University of São Paulo, São Paulo, Brazil
SAMA	South Australian Museum, Adelaide, Australia
SMF	Senckenberg Naturmuseum Frankfurt, Leibniz Institution for Biodiversity and Earth System Research, Frankfurt, Germany
UCMP	University of California Museum of Paleontology, Berkeley, CA, United States of America
USNM	Smithsonian National Museum of Natural History, Washington, DC, United States of America
ZFMK	Zoological Research Museum Alexander Koenig, Leibniz Institute for Animal Biodiversity, Bonn, Germany
ZMB	Museum für Naturkunde, Leibniz Institute for Evolution and Biodiversity Science, Berlin, Germany
ZMPU	Zoological Museum of Perm University, Perm, Russia
ZSM	Zoologische Staatssammlung München, Germany

Table S4. Independent acquisitions of a fully fossorial lifestyle for the here investigated taxa in accordance with stochastic mapping of character evolution (Figure 1). Regarding the higher clade averages, our dataset comprises 85 % of primary and 35 % of secondary acquisitions of a fully fossorial lifestyle. The resulting fully fossorial clusters were used as single converging tips in the convergence analysis.

<table>
<tr><th></th><th>ACQUISITION NO.</th><th>CLUSTER / TAXA INCLUDED</th><th>HIGHER LEVEL</th><th>SAMPLED / TOTAL</th></tr>
<tr><td rowspan="12">primary acquisitions sampled</td><td>1</td><td>Dibamus novaeguineae</td><td>Dibamidae</td><td>1 of 1 (100 %)</td></tr>
<tr><td>2</td><td>Aprasia pulchella</td><td>Gekkota</td><td>1 of 1 (100 %)</td></tr>
<tr><td>3</td><td>Typhlosaurus aurantiacus</td><td rowspan="5">Scincoidea</td><td rowspan="5">5 of 20 (25 %)</td></tr>
<tr><td>4</td><td>Plestiodon reynoldsi</td></tr>
<tr><td>5</td><td>Scincus scincus</td></tr>
<tr><td>6</td><td>Grandidierina petiti</td></tr>
<tr><td>7</td><td>common ancestor of
Saiphos equalis and
Coeranoscincus reticulatus</td></tr>
<tr><td>8</td><td>Bachia panoplia</td><td rowspan="2">non-lacertibaenian
Lacertoidea</td><td rowspan="2">2 of 3 (67 %)</td></tr>
<tr><td>9</td><td>Calyptommatus sinebrachiatus</td></tr>
<tr><td>10</td><td>Amphisbaenia:
Rhineura floridana,
Bipes tridactylus,
Blanus cinereus,
Cadea blanoides,
Trogonophis wiegmanni,
Geocalamus acutus,
Amphisbaena alba,
Amphisbaena cubana,
and Leposternon malkini</td><td>Lacertibaenia</td><td>1 of 1 (100 %)</td></tr>
<tr><td>11</td><td>Anniella pulchra</td><td>Anguimorpha</td><td>1 of 1 (100 %)</td></tr>
<tr><td>12</td><td>common ancestor of
Liotyphlops albirostris and
Afrotyphlops schlegelii</td><td rowspan="10">Serpentes</td><td rowspan="10"><u>primary:</u>
1 of 1 (100 %)

<u>secondary:</u>
9 of 26 (35 %)</td></tr>
<tr><td rowspan="9">secondary acquisitions sampled</td><td>13</td><td>Anilius scytale</td></tr>
<tr><td>14</td><td>Calabaria reinhardtii</td></tr>
<tr><td>15</td><td>common ancestor of
Anomochilus leonardi and
Uropeltis ceylanicus</td></tr>
<tr><td>16</td><td>Achalinus spinalis</td></tr>
<tr><td>17</td><td>Micrelaps vaillanti</td></tr>
<tr><td>18</td><td>Xenocalamus mechowii</td></tr>
<tr><td>19</td><td>Vermicella annulata</td></tr>
<tr><td>20</td><td>Geagras redimitus</td></tr>
<tr><td>21</td><td>Heterodon platirhinos</td></tr>
</table>

Table S5. Per specimen measurements: skull roof compactness (*c*), thickness (*d*), bone overlap (*ovl*), ratio of frontal and parietal (*rfp*), cranial elongation (*elo*), skull diameter (*dia*), and lifestyle (1 = non-fossorial, 2 = semi-fossorial, 3 = fully fossorial)

species	lifestyle	*c* [%]	*d* [%]	*ovl* [%]	*rfp* [-]	*elo* [-]	*dia* [mm]
Sphenodon punctatus	2	87.6	1.79	28.6	1.03	1.38	41.17
Dibamus novaeguineae	3	99.1	1.61	34.9	0.72	2.41	3.05
Lialis burtonis	2	98.1	1.34	32.7	0.69	3.33	8.09
Aprasia pulchella	3	99.0	1.68	16.7	0.75	2.16	2.96
Sphaerodactylus bromeliarum	1	98.3	0.79	33.3	1.10	2.06	3.29
Tarentola mauritanica	1	86.7	1.68	26.7	1.31	1.85	10.53
Gekko gecko	1	75.1	1.67	21.6	1.27	1.60	20.98
Xantusia riversiana	1	94.5	1.57	20.7	0.76	1.90	8.80
Tetradactylus africanus	1	94.7	1.42	22.5	1.02	2.32	6.13
Platysaurus capensis	1	98.1	1.15	25.9	0.97	2.07	8.38
Cordylus cataphractus	1	81.0	1.74	20.1	0.86	1.51	15.06
Chamaesaura anguina	1	92.0	1.62	26.0	0.98	2.47	6.48
Typhlosaurus aurantiacus	3	99.2	1.30	27.1	0.58	2.19	3.86
Plestiodon latiscutatus	2	93.6	1.23	16.5	1.03	1.59	10.35
Plestiodon reynoldsi	3	99.8	1.05	22.1	0.90	2.18	3.28
Scincus scincus	3	95.4	1.49	29.1	1.15	1.98	12.37
Grandidierina petiti	3	99.7	1.09	25.0	0.51	2.45	2.46
Sphenomorphus solomonis	1	99.7	1.21	15.4	0.87	1.90	6.38
Saiphos equalis	3	98.7	1.32	28.9	0.80	2.25	4.67
Coeranoscincus reticulatus	3	99.9	1.56	29.8	0.76	2.32	7.81
Eulamprus quoyii	1	75.6	2.03	22.2	1.06	3.08	7.49
Lerista timida	2	98.7	1.37	38.5	0.86	2.41	2.61
Egernia kingii	2	94.0	1.75	27.9	0.87	1.78	13.57
Tiliqua scincoides	1	80.6	1.38	24.3	1.03	1.97	16.30
Liopholis whitii	2	91.3	1.66	17.8	1.02	1.95	10.61
Eutropis multifasciata	2	90.8	1.21	21.2	1.17	1.95	10.17
Trachylepis quinquetaeniata	1	88.2	1.39	24.2	1.21	1.69	12.17
Kentropyx altamazonica	1	73.6	1.43	28.4	1.35	2.01	11.14
Alopoglossus copii	2	92.7	0.99	33.8	1.24	1.91	8.72
Bachia flavescens	2	100.0	1.20	35.4	1.12	2.46	3.39
Bachia panoplia	3	100.0	1.44	34.6	0.86	2.00	5.47

Calyptommatus sinebrachiatus	3	99.9	0.88	42.6	0.69	2.01	2.88
Gymnophthalmus lineatus	2	99.9	0.91	36.9	0.97	2.15	3.02
Spathorhynchus fossorium	n/a	97.9	2.15	45.5	0.82	1.94	17.85
Dyticonastis rensbergeri	n/a	97.8	2.28	39.0	0.82	2.26	10.41
Hyporhina antigua	n/a	99.7	2.58	37.2	0.72	2.02	5.08
Rhineura floridana	3	99.1	1.92	32.3	0.81	2.21	4.26
Rhineura hatcheri	n/a	96.1	2.24	42.1	0.88	2.37	6.30
Bipes tridactylus	3	95.8	2.22	41.8	0.72	1.79	4.37
Blanus cinereus	3	99.6	1.84	39.4	0.47	2.16	3.10
Cadea blanoides	3	99.8	2.15	46.5	0.49	2.29	5.77
Trogonophis wiegmanni	3	99.9	2.31	45.5	0.35	2.15	5.10
Geocalamus acutus	3	99.7	1.84	43.8	0.71	2.29	3.31
Amphisbaena alba	3	97.8	2.72	41.9	0.54	2.40	9.23
Amphisbaena cubana	3	99.8	2.14	46.9	0.50	2.66	3.44
Leposternon malkini	3	98.9	2.76	46.8	0.69	1.85	8.64
Cryptolacerta hassiaca	n/a	97.4	1.51	18.8	0.82	2.03	8.96
Psammodromus algirus	1	88.3	1.42	30.0	1.09	1.79	9.94
Gallotia stehlini	1	87.5	1.74	23.2	1.14	1.70	18.19
Atlantolacerta andreanskyi	1	89.6	1.16	28.8	0.98	1.91	5.93
Nucras tessellata	2	89.6	1.86	31.5	0.97	1.86	9.47
Gastropholis echinata	1	95.8	1.96	27.4	0.95	2.12	10.51
Acanthodactylus boskianus	2	90.5	1.13	29.2	1.27	1.86	9.12
Phoenicolacerta laevis	1	93.3	1.20	28.0	1.14	2.06	7.78
Lacerta schreiberi	2	88.4	1.30	20.7	1.05	1.89	11.06
Archaeolacerta bedriagae	1	93.9	1.59	26.9	1.15	1.95	8.35
Darevskia mixta	1	88.5	1.03	26.9	1.05	2.35	5.25
Anniella pulchra	3	99.3	1.42	37.1	0.63	2.48	3.70
Diploglossus fasciatus	1	86.9	1.25	28.5	0.89	1.81	12.36
Ophiodes striatus	2	91.5	1.65	21.2	0.93	2.30	8.99
Celestus haetianus	2	95.3	2.35	27.2	0.75	1.97	7.95
Dopasia gracilis	2	99.4	1.39	22.3	0.85	1.85	8.06
Ophisaurus koellikeri	2	99.4	1.40	23.7	0.86	2.04	6.65
Anguis fragilis	2	98.2	1.47	22.9	0.79	2.16	7.01
Gerrhonotus liocephalus	1	86.8	1.78	29.5	0.88	1.60	17.26

Abronia spec.	1	97.4	1.67	35.0	0.95	2.02	7.52
Varanus griseus	2	80.2	1.30	30.7	1.19	1.92	29.33
Varanus bengalensis	2	97.8	1.26	25.8	0.94	2.21	22.32
Furcifer minor	1	70.6	1.00	24.3	1.70	1.47	12.33
Tympanocryptis lineata	1	86.3	1.23	29.1	1.18	1.31	10.33
Phrynocephalus mystaceus	2	79.5	1.67	22.2	0.92	1.08	19.00
Draco volans	1	75.8	1.25	22.1	1.10	1.58	8.41
Uranoscodon superciliosus	1	78.8	1.59	19.8	0.98	1.41	19.42
Leiocephalus carinatus	2	81.2	1.11	21.2	1.06	1.49	12.53
Phrynosoma orbiculare	2	55.8	1.19	12.2	0.98	0.91	20.02
Corytophanes cristatus	1	64.9	1.33	31.6	1.05	1.44	18.46
Anolis sagrei	2	77.5	1.85	27.3	1.22	1.89	8.08
Liotyphlops albirostris	3	99.1	1.68	7.6	0.45	2.64	1.66
Afrotyphlops schlegelii	3	97.4	2.65	24.8	1.53	2.12	4.98
Anilius scytale	3	100.0	1.83	28.4	0.34	2.49	6.83
Calabaria reinhardtii	3	98.6	2.55	7.3	0.67	2.00	10.22
Candoia carinata	1	99.0	1.61	5.3	0.55	2.64	7.94
Eryx conicus	2	99.4	1.78	19.4	0.54	2.24	8.05
Anomochilus leonardi	3	100.0	2.38	32.4	0.38	2.27	3.98
Uropeltis ceylanicus	3	98.9	1.61	29.7	0.52	2.85	4.18
Python bivittatus	1	99.6	1.50	14.9	0.54	2.16	34.63
Achalinus spinalis	3	100.0	1.41	13.0	0.38	2.49	6.67
Cerastes vipera	2	95.3	1.37	5.4	0.65	1.93	10.14
Bothrops lanceolatus	1	94.6	0.93	3.2	0.56	1.92	9.03
Micrelaps vaillanti	3	98.9	2.07	9.6	0.33	3.22	2.43
Psammophis crucifer	1	97.6	1.15	-0.3	0.78	2.61	7.91
Xenocalamus mechowii	3	98.5	4.93	34.7	0.65	2.89	4.45
Lycodryas maculatus	1	99.6	1.39	-1.5	0.60	2.38	7.21
Dendroaspis angusticeps	1	99.9	1.45	-5.6	0.68	2.61	15.64
Acanthophis antarcticus	1	94.3	1.11	6.4	0.57	1.67	13.52
Vermicella annulata	3	97.7	1.88	13.8	0.56	2.64	5.74
Geagras redimitus	3	94.2	1.92	20.9	0.68	2.26	3.48
Lycognathophis seychellensis	1	95.9	1.61	6.6	0.70	2.71	10.16
Heterodon platirhinos	3	98.5	1.89	15.2	0.94	2.40	12.46

Table S6. Key to species name abbreviations used in Figure 4 and S2. Two-letter codes represent fully fossorial tips, three-letter codes represent non- and semi-fossorial tips. Species in italic letters, other groups (in accordance with Table S4) in roman letters.

Abs = *Abronia spec.*	***Dea*** = *Dendroaspis angusticeps*	***Psc*** = *Psammophis crucifer*
Aca = *Acanthophis antarcticus*	***Euq*** = *Eulamprus quoyii*	***Pyb*** = *Python bivittatus*
**Am** = Amphisbaenia	***Fum*** = *Furcifer minor*	**Sc** = common ancestor of *Saiphos equalis* & *Coeranoscincus reticulatus*
Asc = *Anilius scytale*	***Gas*** = *Gallotia stehlini*	
Asp = *Achalinus spinalis*	***Geg*** = *Gekko gecko*	***Spp*** = *Sphenodon punctatus*
Baf = *Bachia flavescens*	***Gel*** = *Gerrhonotus liocephalus*	***Ss*** = *Scincus scincus*
Bol = *Bothrops lanceolatus*	***Gr*** = *Geagras redimitus*	***Tam*** = *Tarentola mauritanica*
Bp = *Bachia panoplia*	***Hp*** = *Heterodon platirhinos*	***Tyl*** = *Tympanocryptis lineata*
**bS** = basal Serpentes	***Kea*** = *Kentropyx altamazonica*	***Urs*** = *Uranoscodon superciliosus*
Cca = *Cordylus cataphractus*	***Lib*** = *Lialis burtonis*	***Vab*** = *Varanus bengalensis*
Ccr = *Corytophanes cristatus*	***Lym*** = *Lycodryas maculatus*	***Vag*** = *Varanus griseus*
Ceh = *Celestus haetianus*	***Mv*** = *Micrelaps vaillanti*	***Vp*** = *Grandidierina petiti*
Cr = *Calabaria reinhardtii*	***Phm*** = *Phrynocephalus mystaceus*	***Xm*** = *Xenocalamus mechowii*
Cs = *Calyptommatus sinebrachiatus*	***Pho*** = *Phrynosoma orbiculare*	
Dam = *Darevskia mixta*	***Pr*** = *Plestiodon reynoldsi*	
