# Supplementary figures and images for: First evidence of convergent lifestyle signal in reptile skull roof microanatomy
Source: BMC Biol. 2020 Nov 30;18:185. doi: 10.1186/s12915-020-00908-y (PMC7702674; doi:10.1186/s12915-020-00908-y)

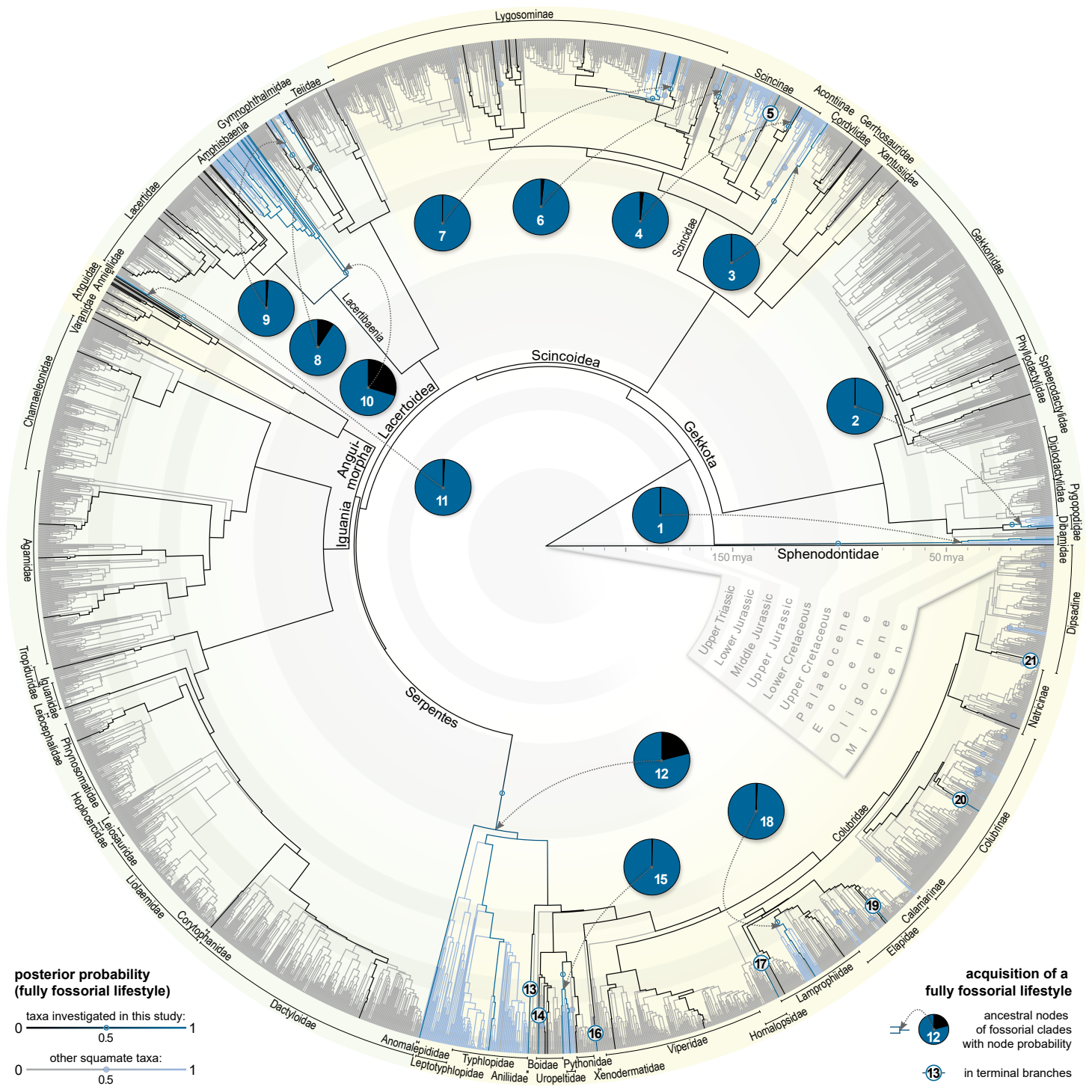

Supplement: Supplementary file 3 — Additional file 3. High resolution version of Fig. 1. [file 12915_2020_908_MOESM3_ESM.pdf]
